# Design and Performance Analysis of Split Ring Resonator-Based Sensor for Soil Moisture Content Characterization

**DOI:** 10.3390/s26175493

**Published:** 2026-08-29

**Authors:** Salman Alduwish, Yongxiang Li, James Scott, Akram Hourani, Nasir Mahmood

**Affiliations:** 1 School of Engineering, RMIT University, Melbourne P.O. Box 3000, Australia; james.scott@rmit.edu.au (J.S.); akram.hourani@unimelb.edu.au (A.H.); 2 School of Science, RMIT University, Melbourne 3001, Australia; nasir.mahmood@rmit.edu.au

**Keywords:** soil moisture sensors, split ring resonator (SRR), complex permittivity, sandy and loamy soils, machine learning calibration

## Abstract

This paper addresses the need for compact, low-cost soil moisture sensors operating at low microwave frequencies that can provide accurate, texture-aware (i.e., sensitive to different soil particle-size distributions such as sand and loam) characterization of soil permittivity and moisture content. Conventional techniques and many existing microwave resonator sensors are constrained by limited penetration depth, relatively large or complex structures, and calibration procedures that do not robustly account for different soil textures and moisture ranges. A dual-port microstrip square split ring resonator (SRR) sensor on Rogers RO3010 (Rmit University, Melbourne, Australia) is designed for operation at 1.3 GHz and analyzed using full-wave 3D electromagnetic simulations. The structure employs a T-shaped feedline and a shunt quarter-wavelength matching section to achieve strong field confinement in the sensing region and effective impedance matching. Soil is modeled as sandy and loamy superstrates over practical agricultural moisture ranges, with their complex permittivities drawn from reference datasets. Empirical calibration models are then developed, including polynomial curve fitting between resonance frequency shift and real permittivity, machine-learning-based calibration using resonance frequency and transmission loss features, and multiple linear regression linking moisture content to both real and imaginary permittivity components. The sensor exhibits a resonance frequency shift of about 115 MHz over 0–30% moisture for sand and 0–40% for loam, with a maximum sensitivity of 3.4%. Calibration models achieve mean absolute error below 1.22%, root mean square error under 1.58%, and coefficients of determination R^2^ > 0.98 for both soil textures. These results demonstrate that a compact 1.3 GHz square SRR sensor with data-driven calibration, i.e., empirical models learned from simulated and measured S-parameters, enables sensitive, reproducible, and texture-aware soil moisture estimation suitable for agricultural and environmental monitoring.

## 1. Introduction

Accurate measurement of soil moisture content (MC) is essential for optimizing irrigation, improving crop yield, and monitoring hydrological and environmental processes. Reliable MC data underpin precision agriculture, drought assessment, and climate models, driving a growing demand for sensing technologies that are fast, low-cost, and deployable in the field.

Conventional soil moisture measurement techniques, such as gravimetric analysis, time-domain reflectometry (TDR), and capacitance probes, can provide good accuracy but often suffer from drawbacks including destructive sampling, high instrumentation cost, complex calibration, or limited suitability for dense spatial monitoring. Microwave resonance sensors (MRSs) have emerged as an attractive alternative due to their ability to infer MC from soil dielectric properties, offering rapid, potentially non-contact or minimally invasive measurements with simple hardware implementation [1,2,3]. Various MRS configurations based on antennas and resonators have been investigated, showing that resonator-based designs, in particular, can achieve high resolution and sensitivity without requiring direct electrode–soil contact [4,5,6,7,8,9,10,11,12,13,14,15,16,17,18,19,20].

Among resonator-based approaches, split ring resonators (SRRs) have demonstrated strong field confinement and high sensitivity to permittivity changes, making them suitable for dielectric characterization of materials including soils [13,14,16,17,18,19,20]. Existing SRR- and planar-resonator soil moisture sensors, however, are predominantly designed at higher microwave frequencies and often present several limitations. These include reduced penetration depth in soil, relatively large footprints or complex geometries that hinder integration into compact sensor networks, and calibration procedures that do not robustly link scattering parameters to both real and imaginary parts of soil permittivity, nor explicitly address differences across soil textures and moisture ranges [21,22,23,24,25,26,27,28,29,30,31,32,33]. Consequently, there remains a need for compact, low-frequency microstrip SRR sensors with well-founded calibration models that can accurately and reproducibly quantify soil permittivity and MC for different soil types in practical agricultural ranges.

This work addresses this gap by designing and numerically evaluating a compact microstrip square split ring resonator (SRR)-based sensor optimized to operate at 1.3 GHz for soil moisture monitoring. Specifically, the study aims first to design and optimize a dual-port square SRR sensor on Rogers RO3010, incorporating a T-shaped feedline and a shunt quarter-wavelength matching section, and to characterize its baseline resonance performance using full-wave 3D simulations. It further aims to investigate the sensor’s response to soil superstrates representing sandy and loamy textures over relevant MC ranges, analyzing resonance frequency shifts and transmission loss as functions of complex permittivity. In addition, the study seeks to develop and validate empirical calibration models that map measured scattering responses to soil permittivity and MC, using polynomial curve fitting between frequency shift and real permittivity, machine-learning-based calibration from frequency and transmission features, and multiple linear regression linking MC to both the real and imaginary parts of permittivity. Finally, the work aims to evaluate model accuracy and overall sensor performance through error analysis in terms of mean absolute error (MAE), root mean square error (RMSE), and coefficient of determination (R^2^), and to benchmark the proposed sensor against existing microwave soil moisture sensors with respect to operating frequency, size, sensitivity, and applicable moisture range. Through these objectives, the paper tests the hypothesis that a compact, low-frequency square-SRR microstrip sensor, combined with data-driven calibration models, can provide sensitive, texture-aware, and quantitatively accurate soil moisture characterization suitable for practical agricultural monitoring.

In contrast to most SRR- and planar-resonator soil moisture sensors that operate at higher microwave frequencies with reduced penetration depth and often ad hoc calibration for a single soil texture, the proposed work makes three specific advances. First, it demonstrates a compact square SRR sensor optimized at 1.3 GHz, improving field penetration while maintaining a small 26 × 43 mm footprint suitable for dense sensor deployments. Second, it develops a calibration framework that jointly exploits resonance frequency shift and transmission loss, and explicitly links both the real and imaginary parts of soil permittivity to moisture content via polynomial fitting, machine-learning regression, and multiple linear regression. Third, the study provides texture-aware calibration by deriving separate models for sandy and loamy soils using reference permittivity datasets and validating these models with experimental measurements, thereby addressing soil texture dependence that is only partially treated in previous microwave resonator sensors. Together, these contributions provide a low-frequency, compact, and data-driven sensing approach that more comprehensively addresses penetration depth, footprint, calibration robustness, and texture coverage than earlier SRR and planar resonator designs.

To address the above-mentioned gap and objectives, this research paper is organized as follows. Section 2 focuses on the split ring resonator-based sensor, and is divided into two parts: the first explains the applied design methodology from initial specifications to the final sensor configuration; the second presents an analysis of the simulated electromagnetic responses. Section 3 introduces the soil measurement properties and describes how the designed sensor is used for soil moisture characterization. Section 4 develops empirical models to retrieve soil permittivity and moisture content from the sensor’s scattering responses, including polynomial curve fitting, machine-learning-based calibration, and multiple linear regression. Section 5 focuses on experimental validation of the designed sensor with the soil. Section 6 discusses the regression models, error analysis, and a comparison of the proposed sensor with related work in the literature. The final section provides the conclusion and summarizes the major contributions of this work.

## 2. Split Ring Resonator-Based Sensor: Methodology and Design

**Figure 1:**
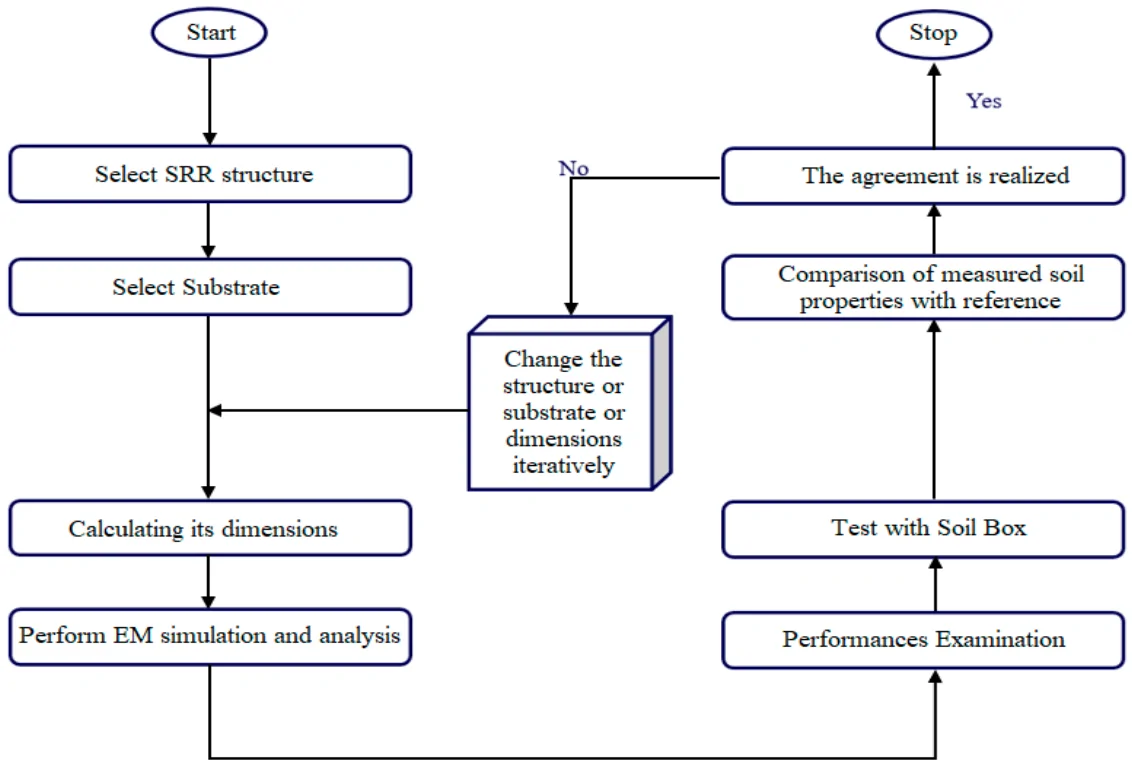
Flow-chart of the sensor design procedure.

The design and development of the split ring resonator (SRR)-based sensor for soil analysis follows a systematic procedure comprising two main stages, as illustrated in Figure 1.

**Figure 2:**
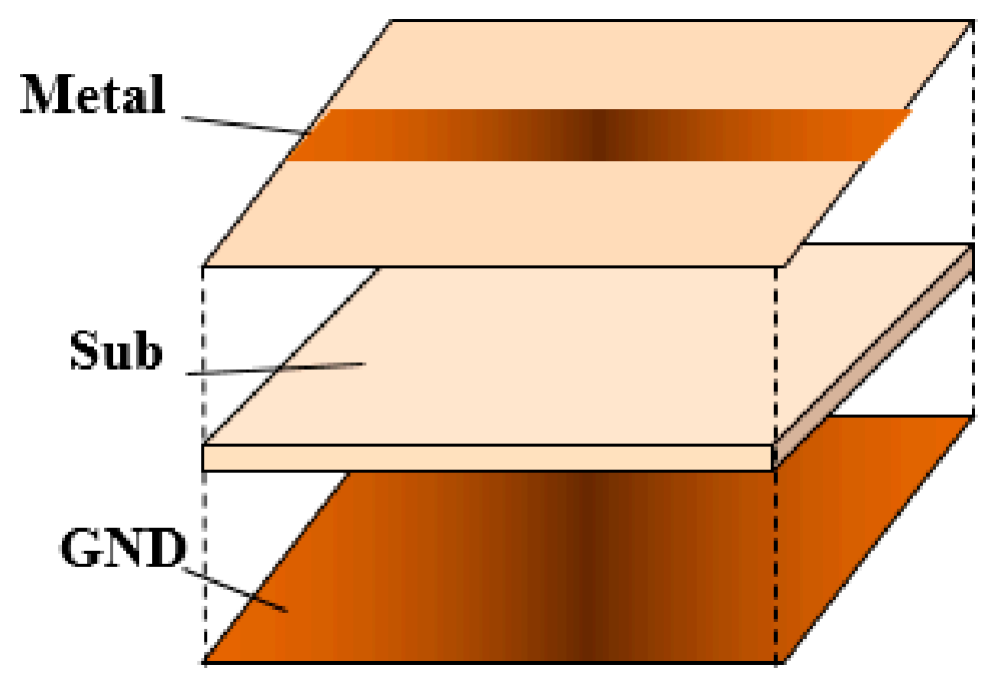
Stack-up of microstrip technology.

The first stage focuses on defining the sensor specifications, performing electromagnetic (EM) design using microstrip technology, and evaluating the performance of the SRR structure (Figure 2). In this stage, the resonator geometry and dimensions are optimized through full-wave EM simulations to achieve the desired resonance frequency, impedance matching, and field distribution. The resulting performance metrics are then used to fine-tune the design parameters to improve sensitivity, selectivity, and overall stability of the sensor.

The second stage involves testing of the optimized sensor with soil samples to assess its performance. In this phase, the sensor is exposed to different soil types and moisture levels to evaluate its response under realistic conditions. The measured scattering parameters are analyzed to verify the sensor’s sensitivity and accuracy in detecting changes in soil dielectric properties. This stage may require several iterations of measurement and refinement to ensure that the fabricated sensor performance aligns with the simulation results and meets the application requirements. Detailed descriptions of the soil types, sample preparation, and measurement protocol are provided in Section 3, Section 4 and Section 5.

**Figure 3:**
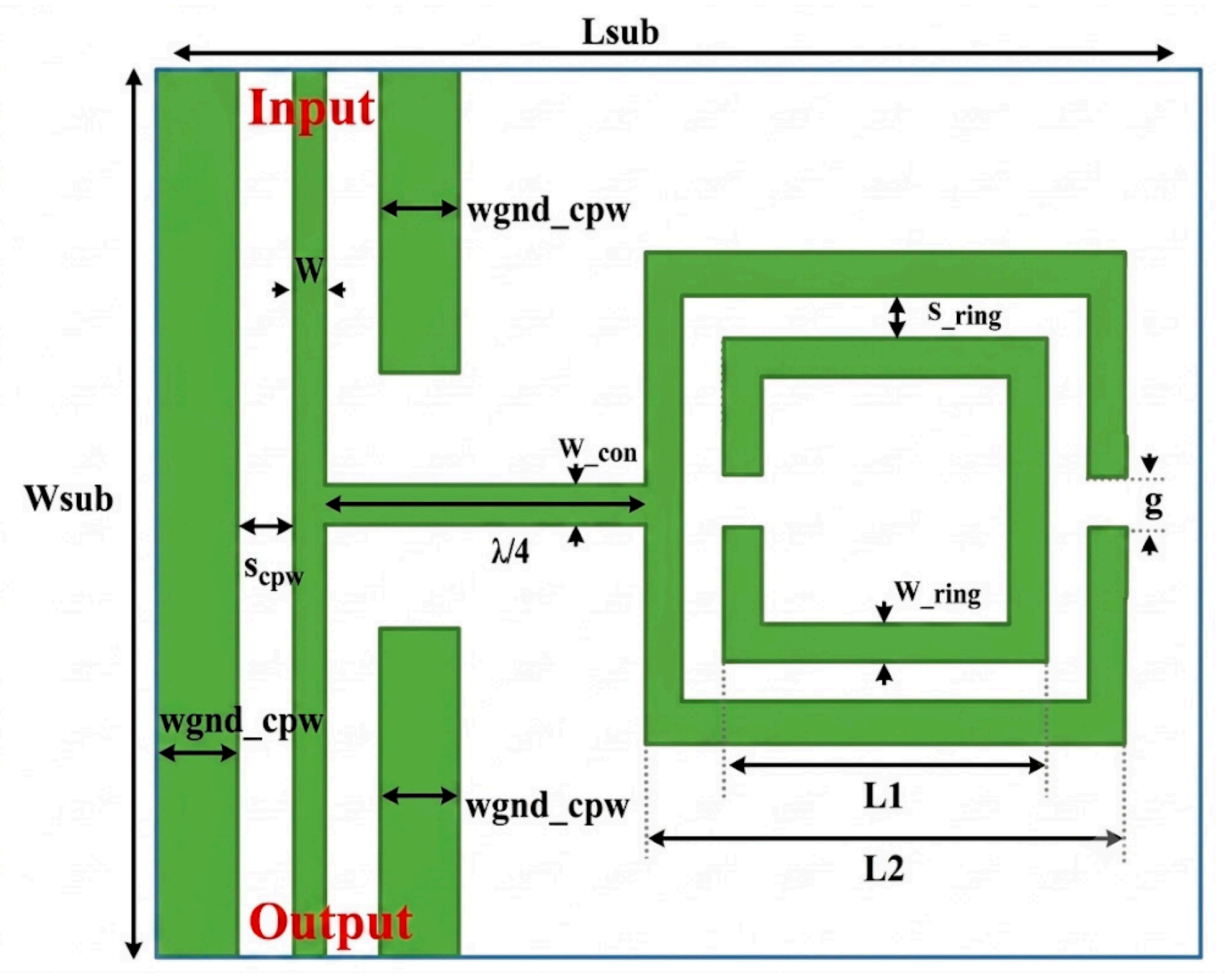
Configuration of square SRR-based sensor.

The split-ring topology-based dual-port sensor is designed to characterize the dielectric properties of the soil in terms of moisture content. The proposed sensor is composed of three layers: the top and bottom layers are made of conductive copper material with a thickness of 35 µm, and the middle layer is made of a dielectric substrate (Rogers RO3010) to provide structural support, with a relative permittivity of 11.2 and a thickness of 1.27 mm. A square-shaped SRR is adopted because it offers a compact footprint, a well-defined resonant response, and strong field confinement in the sensing region, which enhances sensitivity to variations in soil permittivity while remaining compatible with standard planar fabrication. The SRR is connected to the input and output ports via a T-shaped feedline, as illustrated in Figure 3. The corresponding design parameters are detailed in Table 1.

The proposed structure is optimized for an operating frequency of ~1.3 GHz, which was selected as one of three representative frequencies for analyzing the dielectric properties of soils [27]. This choice is based on the measurement dataset and reflects the fact that lower microwave frequencies are more relevant for soil characterization due to their higher penetration depth, while still offering a practical compromise between sensor compactness, sensitivity, and measurement reliability. At 1.3 GHz, the guided wavelength is sufficiently small to realize a compact quarter-wavelength (λ/4) microstrip line and split ring resonator (SRR), while the corresponding line widths and gaps remain compatible with standard PCB fabrication tolerances and exhibit acceptable conductor and dielectric losses. The sensor design employs a quarter-wavelength (λ/4) microstrip line connected in shunt with the transmission line, with a width of 0.93 mm in all cases, as calculated from (Equation (1)). In this configuration, the λ/4 microstrip line functions as an impedance-matching element between the feedline and the split ring resonator (SRR). The chosen SRR geometry enhances the electromagnetic field at the resonance frequency, providing strong field confinement with sufficient penetration into the material under test, thereby improving the sensor sensitivity.

The dual-port T-shaped feedline, together with the shunt quarter-wavelength matching section, is specifically designed to (i) provide effective impedance matching between the external ports and the SRR at 1.3 GHz, and (ii) concentrate the electric field in the vicinity of the SRR gap where the soil superstrate is placed. This configuration enhances the coupling between the resonant fields and the material under test while preserving a compact footprint.

The total length of the SRR remains fixed at λ/2 (excluding the split ring gap g), where λ is the wavelength in the guided medium. The length of the outer split ring is obtained from Equation (Equation (3)), and the value of the guided wavelength λ can be calculated using Equation (Equation (4)) [13].

(1) $$W=\frac{2}{\pi }h_{sub}\left[\left(B-1\right)-\ln(2B-1)+\frac{\varepsilon_{r}-1}{2\varepsilon_{r}}\left[\ln(B-1)+0.39-\frac{0.61}{\varepsilon_{r}}\right]\right]$$

(2) $$B=\frac{60\pi^{2}}{Z_{c}\sqrt{\varepsilon_{r}}}$$

(3) $$L=\frac{\lambda }{2}=\frac{\lambda_{0}}{2\sqrt{\varepsilon_{eff}}}$$

(4) $$\lambda =\frac{c}{f_{0}}$$

where “*L*” is the length of the metal track, “*W*” is its width, “h_Sub_”, “*ε_r_*”, “*ε_eff_*” are respectively, the height, dielectric permittivity of the substrate, and the effective relative permittivity of the microstrip line.

The SRR and feedline dimensions were optimized through parametric sweeps in CST Microwave Studio to satisfy three main criteria: (i) a resonance centered at 1.3 GHz under unloaded conditions; (ii) (|S11| < −20 dB) at resonance for good impedance matching; and (iii) strong electric-field confinement in the sensing region above the SRR gap while maintaining acceptable conductor and dielectric losses. The key geometric parameters, including the inner and outer ring side lengths, ring width, gap, and spacing, as well as the width and length of the quarter-wavelength matching line, were varied within fabrication-compatible ranges. For each parameter set, the resonance frequency, (|S11|, |S21|), and field distribution were extracted, and the final geometry listed in Table 1 was selected as the best compromise between the above criteria.

**Figure 4:**
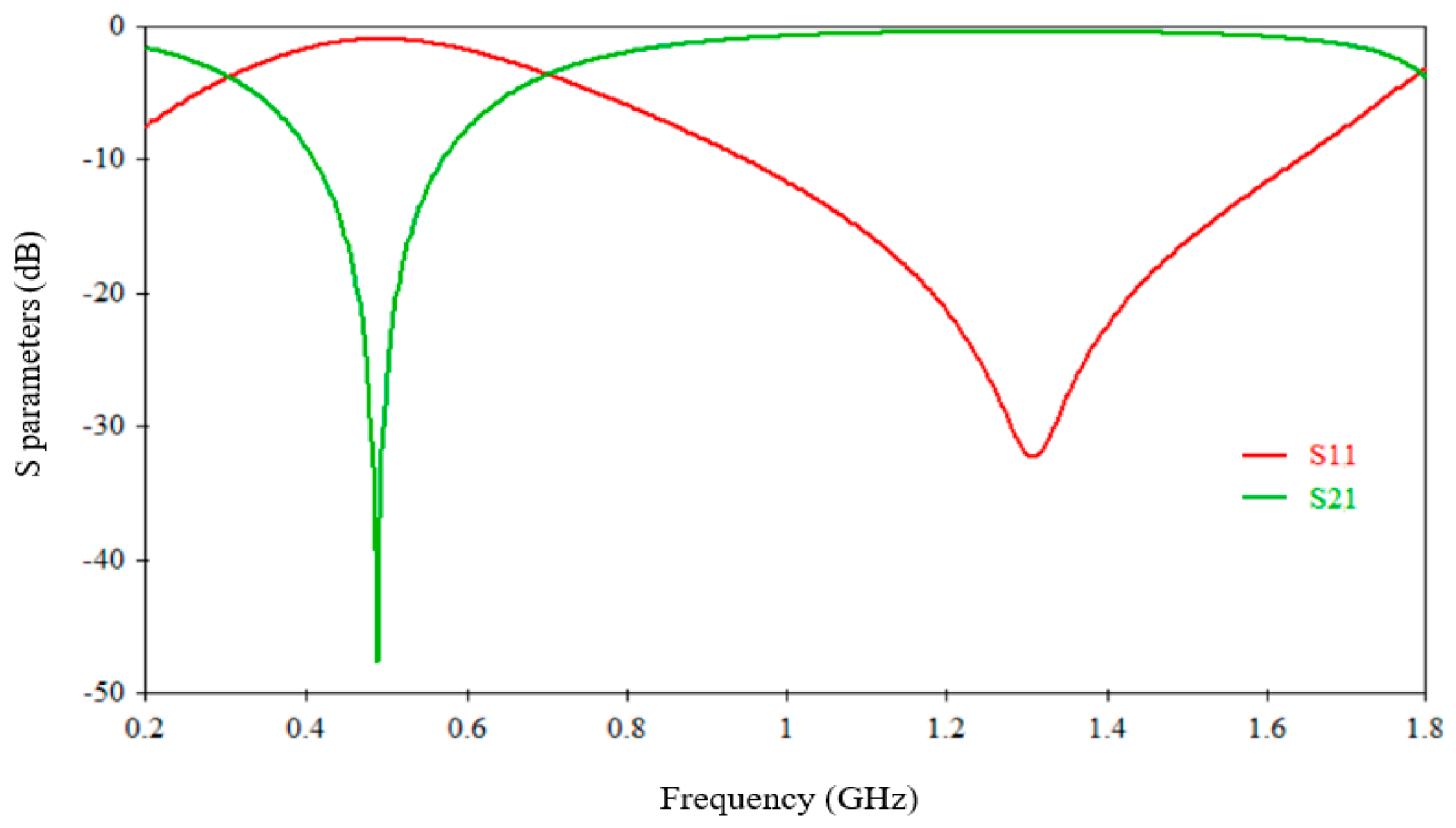
Simulated |S_11_| and |S_21_| response of the proposed square SRR-based sensor.

Figure 4 shows the reflection (S_11_) and transmission (S_21_) response of the square SRR-based sensor. The proposed sensor resonates at 1.3 GHz with |S_11_| = −32.36 dB and |S_21_| = −47.69 dB at frequency 0.49 GHz.

The microwave planar sensor operates as a resonant system whose resonant frequency shifts when its electromagnetic field interacts with a dielectric medium such as soil. In the proposed design, a square SRR-based sensor is used to probe the soil’s dielectric properties by placing the soil as a superstrate on top of the resonator. As described in Equation (Equation (3)), introducing soil with a higher relative permittivity alters the effective permittivity seen by the SRR, which in turn moves the resonant frequency. By accurately measuring and analyzing this frequency shift, the dielectric properties of the soil sample can be inferred. The square-SRR geometry is particularly suitable for this purpose because it combines compact dimensions, high sensitivity to permittivity changes, and straightforward fabrication.

## 3. Square SRR-Based Sensor for Soil Moisture Characterization

**Figure 5:**
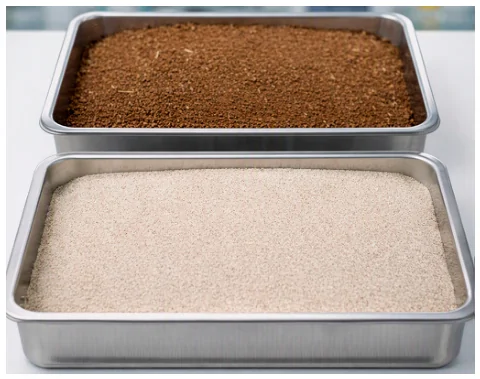
Soil samples.

Soil strength is determined by its solid component, primarily influenced by soil texture and organic matter content. The two main categories of soil texture, based on particle size, are sand and loam (Figure 5). When sand and loam are present in the appropriate proportions, along with adequate organic content, soil quality is enhanced, facilitating the formation of soil aggregates.

The soil’s moisture-holding capacity is reflected by the stability of its aggregates, which depends on the distribution of soil particle sizes; soils with a higher proportion of larger particles are more prone to aggregate disintegration as MC changes. The relative permittivity of dry soil typically falls within the range of 2–3, whereas adding water to the soil significantly increases this value. To gain a better understanding of the dielectric behavior of soil, it is useful to consider the complex relative permittivity, which comprises a real part (dielectric constant) and an imaginary part proportional to energy loss, and is expressed as follows [1]:

(5) $$\varepsilon_{r}=\varepsilon^{\prime }-j\varepsilon^{\prime \prime }$$

where ε′ is the real part of the complex relative permittivity, and ε″ is the imaginary part of the complex relative permittivity.

Variations in particle size and moisture content (MC) cause the dielectric properties of soil to change dynamically. Soil grain size strongly influences these dielectric properties. Sand grains have a lower specific surface area and adsorb less moisture, whereas loam grains, with their higher surface area, adsorb more water through adhesion at the particle surface. Consequently, sandy soils contain a smaller fraction of bound water than loamy soils.

Table 2 shows the estimated microwave properties of sandy soil and loamy soil at 1.3 GHz, based on earlier measurements [27]. These properties, which vary with moisture levels, are incorporated into the 3D numerical model to simulate real conditions of sandy and loamy soil with different moisture levels.

**Figure 6:**
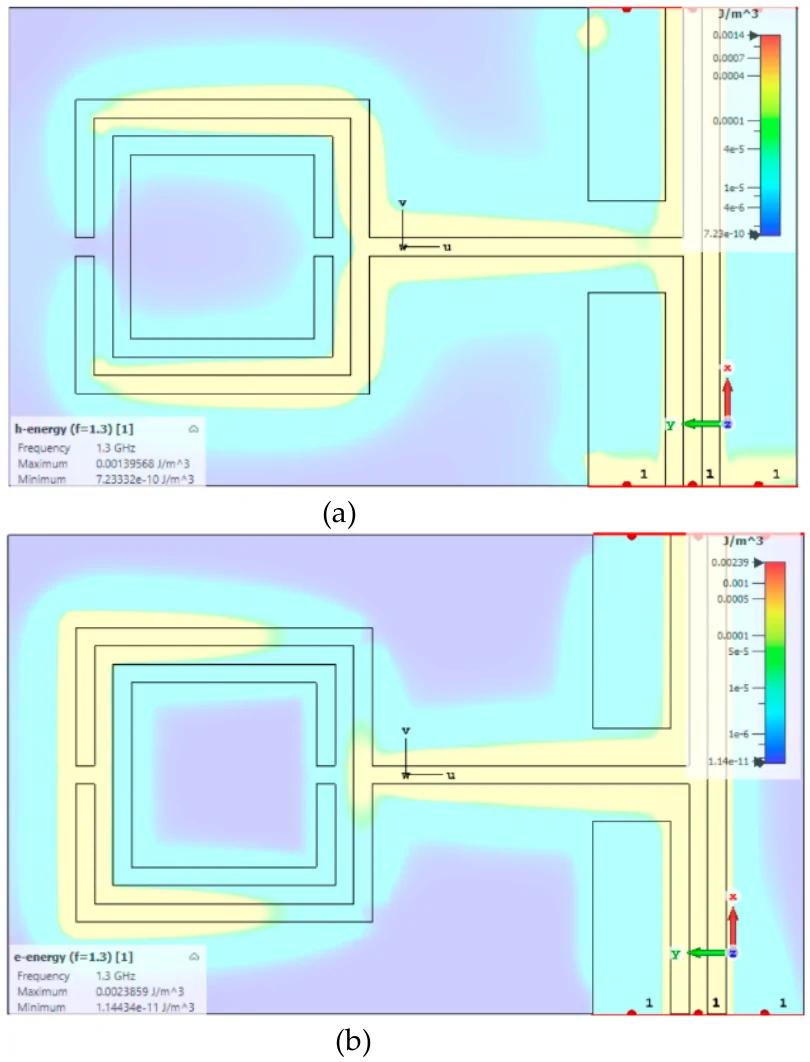
EM field distribution of the proposed square SRR-based sensor: (**a**) magnetic energy, and (**b**) electric energy.

To accurately test the designed sensor, the Sample Under Test (SUT) of soil is positioned in the region with the highest electric field concentration, as shown in Figure 6. This strategic placement ensures optimal interaction between the sample and the electric field, providing the most accurate results for the sensor’s performance evaluation.

**Figure 7:**
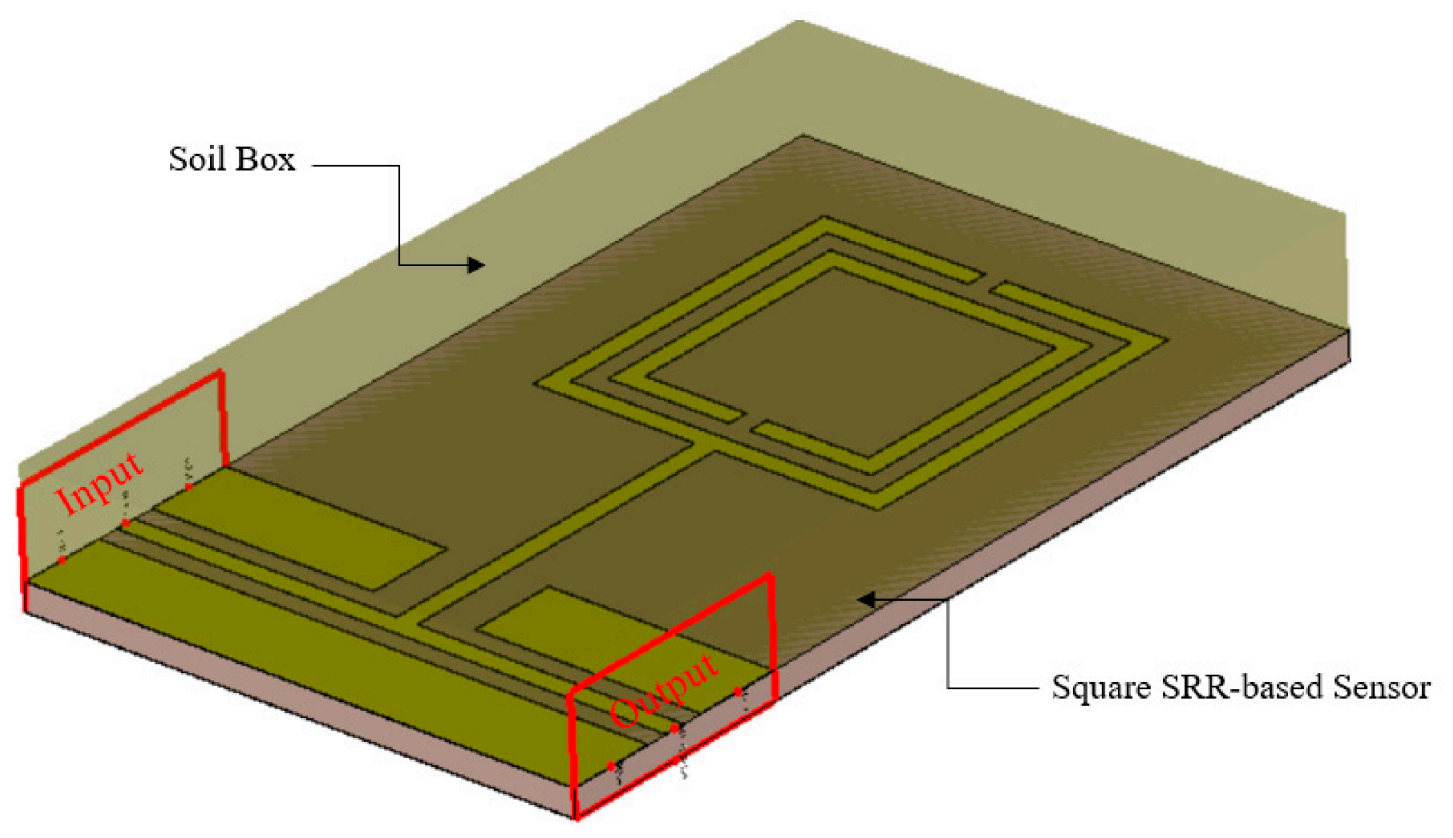
3D view of square SRR-based sensor with soil box.

The soil box was placed above the square SRR-based sensor as a superstrate for loading the soil, as shown in Figure 7. The width and length of the soil box were the same as those of the ground plane of the sensor, and its depth was chosen to be 5 mm. The square SRR-based sensor detects soil moisture by measuring the transmission loss (S21). To ensure the reproducibility and repeatability of the proposed soil moisture square SRR-based sensor, several samples are used for each moisture variation.

In all simulations and measurements, the soil acts as a superstrate that is in direct contact with the upper surface of the substrate above the SRR region; no intentional air gap is introduced between the soil and the sensor. During experiments, the soil box was gently overfilled and then leveled with a flat edge to promote intimate contact and minimize residual air pockets at the soil–sensor interface. The vertical distance between the main soil body and the resonator is therefore determined by the fixed thickness of the soil box wall and the planar sensor surface, resulting in a well-defined and repeatable coupling condition for all measurements.

**Figure 8:**
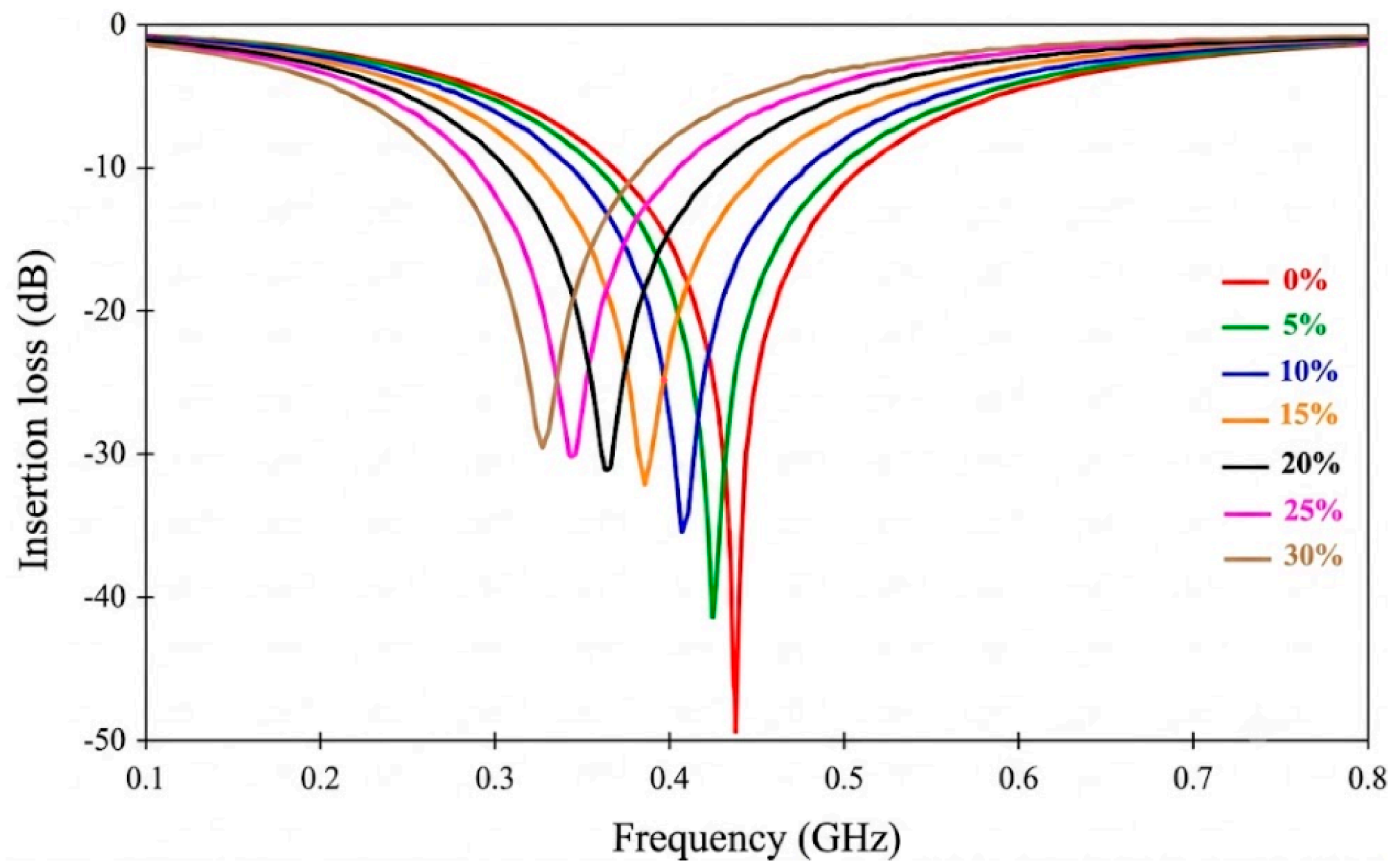
Simulated S_21_ response of square SRR-based sensor with sandy soil.

**Figure 9:**
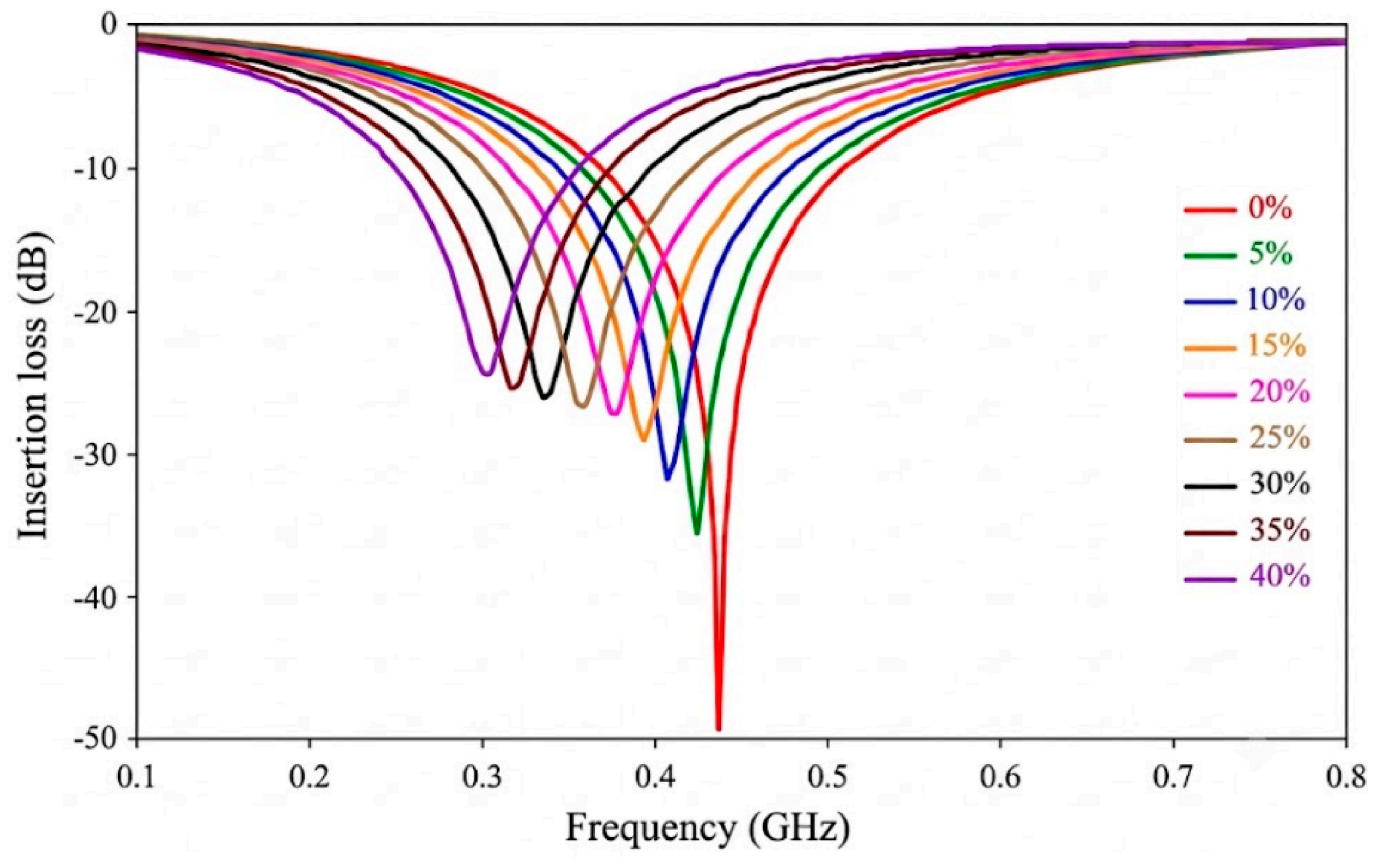
Simulated S_21_ response of square SRR-based sensor with loamy soil.

Figure 8 and Figure 9 illustrate the correlation between moisture content (MC) variations and frequency shifts for sandy and loamy soils, respectively. A frequency shift of approximately 115 MHz was observed across the MC range of 0% to 30% for sandy soil and 0% to 40% for loamy soil. The frequency displacement at each moisture level indicates an increase in relative permittivity corresponding to increased MC.

The observed frequency shift demonstrates the sensor’s sensitivity to changes in soil moisture content. This relationship between moisture content and frequency displacement can be utilized to develop a calibration curve for accurate soil moisture measurements. The proposed square SRR-based sensor shows promise as a reliable and cost-effective tool for monitoring soil moisture in agricultural and environmental applications.

Regarding the transmission response of the proposed SRR-based sensor with varying MC in soil samples, it can be seen that as the MC increases, the frequency shifts toward a lower value, with different soil textures (sand and loam) exhibiting different shifts. It is worth noting that the soil exhibits similar characteristics in its dry form, i.e., at MC = 0% (red curve).

To evaluate the performance of the designed square SRR-based sensor, it is essential to analyze its sensitivity, defined here as the relative change in resonant frequency per unit change in moisture content. This involves obtaining the variation in resonance frequency, which allows us to determine the sensor’s sensitivity.

(6) $$S_{n}=\frac{{\Delta f}_{notch}}{f_{notch\_empty}\times \left(\varepsilon^{\prime }-1\right)}\times 100\%$$

where ${\Delta f}_{notch}$ represents the notch frequency of the proposed MW sensor under loaded conditions (with the soil box). $f_{notch\_empty}=0.4762\;\mathrm{GHz}$.

The sensitivity performance of the proposed square SRR-based sensor was determined from the simulation results presented in Table 3 and Table 4.

## 4. Empirical Modeling of Soil Permittivity Measurement

A numerical model is required to relate the permittivity of the tested soils to the measurable physical parameter provided by the sensor, namely the resonance frequency shift. Using curve-fitting techniques, an empirical equation for the material permittivity is extracted directly from the measured scattering data.

For any given moisture content (MC) level, the absolute prediction error between the measured permittivity and the model output is calculated using (Equation (7)).

(7) $$Error\;\left(\%\right)=\left|\frac{{\varepsilon^{\prime }}_{simulated}-{\varepsilon^{\prime }}_{Dataset}}{{\varepsilon^{\prime }}_{Dataset}}\right|\times 100$$

### 4.1. Polynomial Curve Fitting

Polynomial regression was adopted to model the relationship between the real part of the soil permittivity and the resonance frequency shift in the SRR-based sensor for different moisture content levels. Separate polynomial models were obtained for sand and loam soils to account for their different textural and dielectric properties.

**Figure 10:**
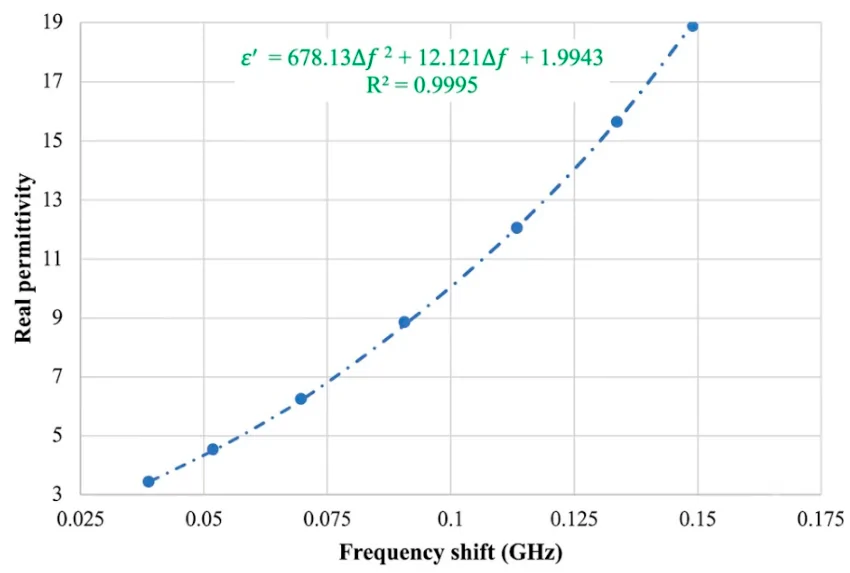
Polynomial fitting model for real permittivity vs. frequency shift in sand soil.

Figure 10 shows the polynomial fitting model for the real permittivity of sand as a function of the resonance frequency shift for all investigated MC levels. The plotted points correspond to the simulated data, while the continuous curve represents the fitted polynomial. The good visual agreement between the data and the fitted curve indicates that the chosen polynomial order is sufficient to capture the nonlinear dependence of permittivity on frequency shift.

Table 5 summarizes the polynomial fitting results for sand soil at each MC level. For each moisture content, the table lists the dataset center frequency in GHz, the simulated permittivity, and the corresponding simulated prediction error obtained from the polynomial model. The relatively low prediction errors over the full MC range (0–30%) confirm the suitability of the polynomial approach for sand soil characterization.

**Figure 11:**
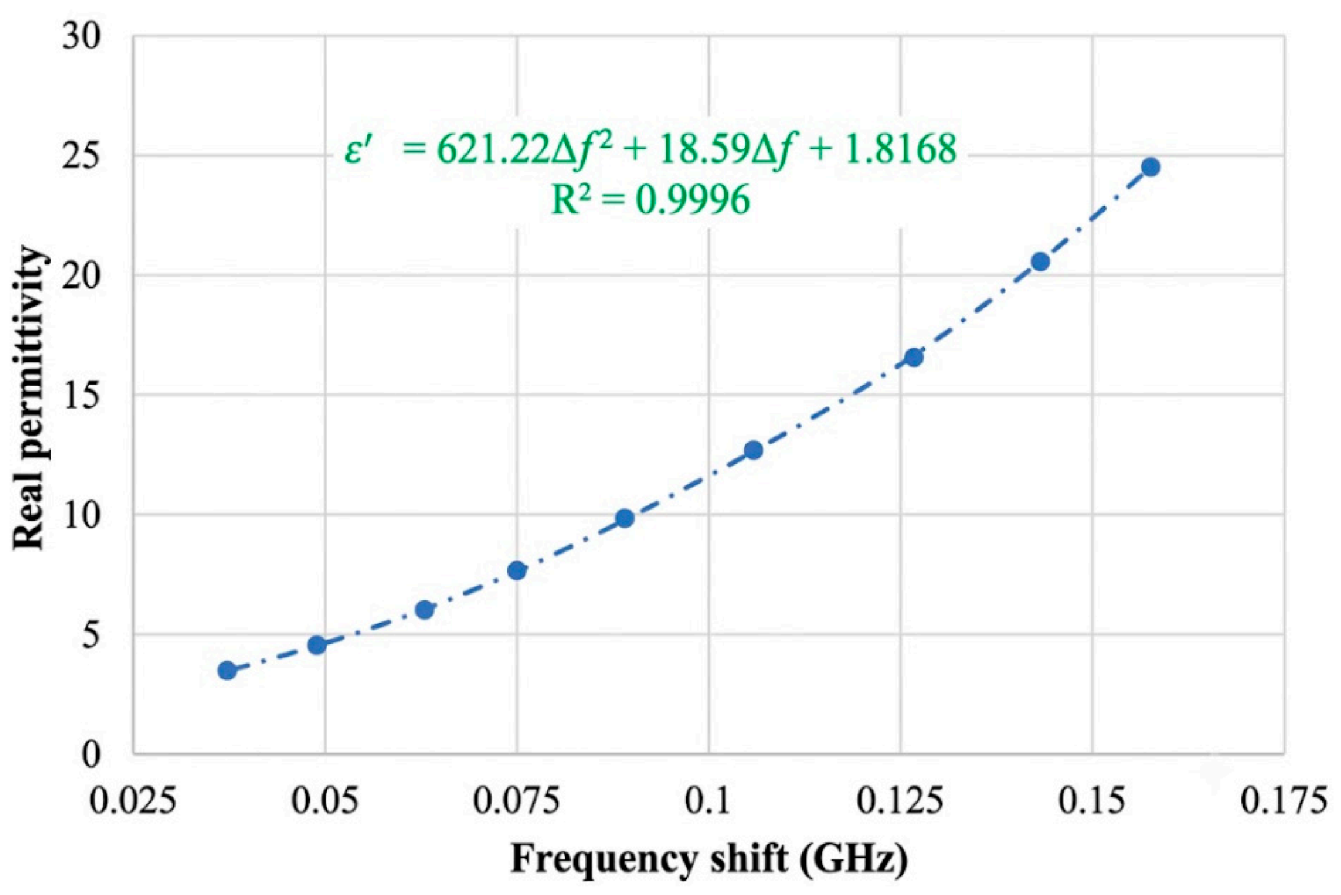
Polynomial fitting model for real permittivity vs. frequency shift in loam soil.

Figure 11 presents the polynomial fitting model for the real permittivity of loam soil versus the resonance frequency shift. As in the sand case, the discrete markers denote the simulated data and the solid line indicates the fitted polynomial. The curve closely follows the simulated points over the whole frequency range, demonstrating that the polynomial model also adequately describes the loam soil behavior.

Table 6 reports the polynomial fitting results for loam soil at each MC level. It includes the dataset center frequency in GHz, the simulated permittivity, and the simulated prediction error for the model. The errors remain acceptably small across the 0–40% MC range, confirming that polynomial fitting provides an accurate and compact empirical model for loam soil permittivity as a function of the measured frequency shift.

### 4.2. Machine Learning Calibration

In addition to polynomial fitting, a data-driven machine learning (ML) calibration procedure was developed to directly map the sensor’s scattering responses to soil permittivity and MC. The ML models use both the resonance frequency shift and the transmission loss as input features, thereby exploiting more information from the measured S-parameters than frequency alone.

**Figure 12:**
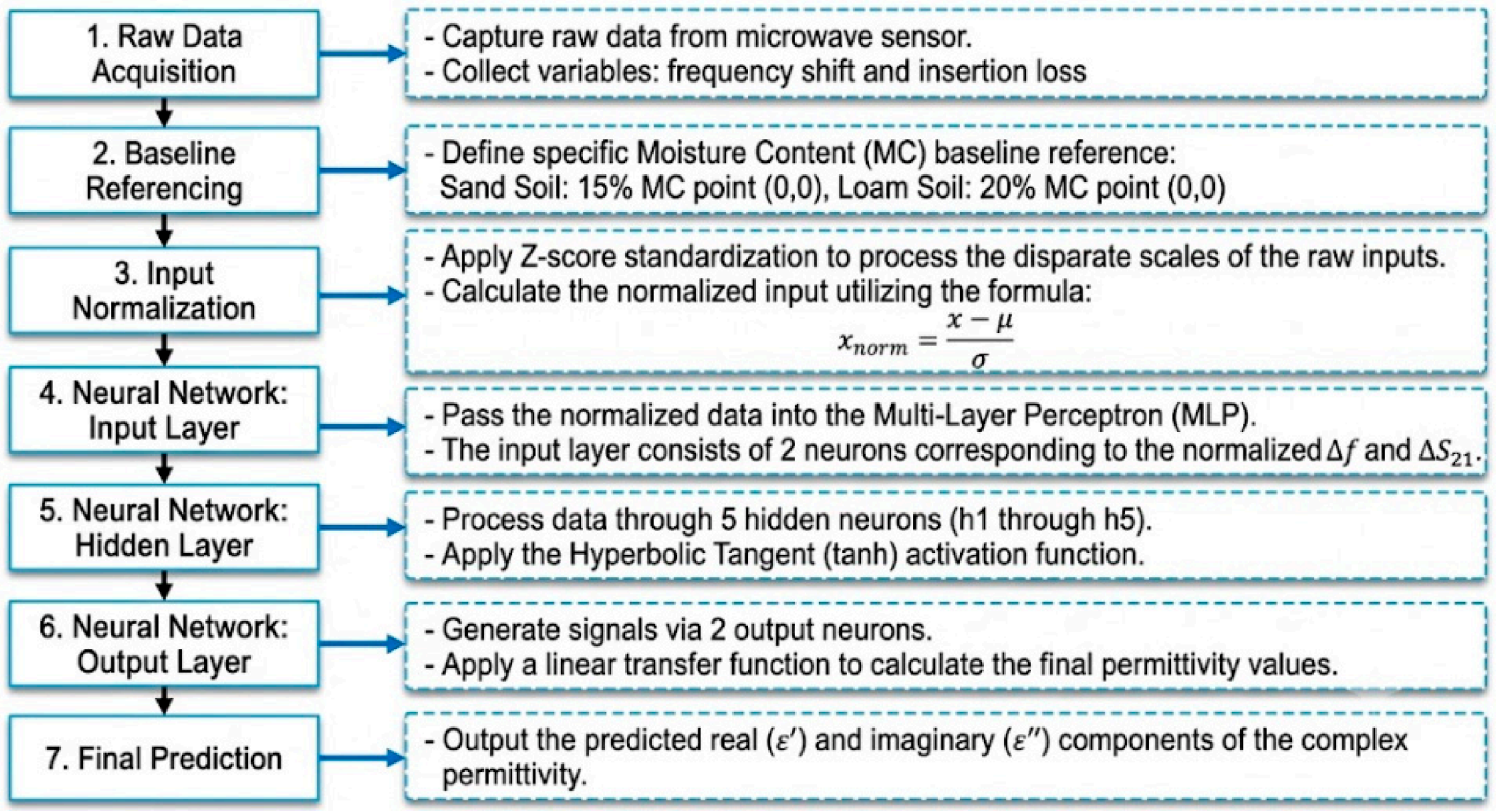
Flow-chart of the machine-learning-based calibration procedure.

Figure 12 shows the overall flow-chart of the ML-based calibration procedure. The process starts from the simulated S-parameter responses of the SRR-based sensor under different soil types and moisture levels. The relevant features (resonance frequency and S21 magnitude at resonance) are then extracted and standardized before being fed into a trained regression network. The network outputs the predicted dielectric properties and, subsequently, the moisture content. Finally, the predictions are compared with the reference values to evaluate the calibration performance and refine the model if needed.

Table 7 lists the standardization parameters used for sand and loam soils. For each soil type and input feature, the mean and scale (standard deviation) values are given. These parameters are used to normalize the raw input features to zero mean and unit variance prior to inference, ensuring numerical stability and consistent performance across different measurement ranges.

Table 8 and Table 9 summarize the network equations and the corresponding prediction equations for sand and loam soils, respectively. The “Network Equations” in Table 8 describe the internal linear and nonlinear transformations applied by the trained model to the standardized input features, whereas the “Prediction Equations” in Table 9 provide the final closed-form expressions used to compute the target variables (e.g., permittivity or MC) from the standardized inputs. These explicit equations enable straightforward implementation of the ML calibration in firmware or embedded systems without requiring a full ML framework.

Table 10 and Table 11 present the ML model results for sandy and loamy soils. Each table reports, for every MC level, the normalized input features (standardized frequency shift and S21), the corresponding ML-predicted values, and the prediction error. For sand soil (Table 10), the predicted MC values closely follow the actual moisture levels across the 0–30% range, with very small absolute errors at most operating points. Similarly, for loam soil (Table 11), the model maintains accurate predictions over the broader 0–40% MC range, again with low deviations from the reference values.

Overall, Figure 12 and Table 7, Table 8, Table 9, Table 10 and Table 11 demonstrate that the proposed ML-based calibration effectively captures the nonlinear relationship between the SRR sensor response and soil properties. By combining frequency and transmission loss features with appropriate standardization and regression modeling, the ML calibration achieves accurate and robust moisture estimation for both sandy and loamy soils, complementing the polynomial curve-fitting approach described in Section 4.1.

Given the limited number of calibration points available from the reference permittivity datasets, the ML models were trained on small simulated datasets linking resonance frequency and (|S21|) magnitude to permittivity and MC. The resulting performance metrics (e.g., MAE, RMSE, (R^2^)) therefore characterize the goodness of fit on these calibration data rather than independent validation accuracy. When the trained models are applied to the experimental S-parameter measurements in Section 5, they qualitatively preserve the expected monotonic dependence of permittivity and moisture content on resonance frequency and transmission loss. However, a larger experimental dataset covering more soil batches and moisture levels will be required in future work to quantitatively assess prediction bias, variance, and potential overfitting under real soil conditions.

### 4.3. Regressing Modeling for Soil Moisture Content

We specify the multiple linear regression model as:

(8) $${MC}_{i}=\beta_{0}+\beta_{1}{\varepsilon_{i}}^{\prime }+\beta_{2}{\varepsilon_{i}}^{\prime \prime }$$

where ${MC}_{i}$ is the moisture content for observation *i*, ${\varepsilon_{i}}^{\prime }$ and ${\varepsilon_{i}}^{\prime \prime }$ are the real and imaginary permittivity values, respectively, $\beta_{0}$ is the intercept, and $\beta_{1}$ and $\beta_{2}$ are regression coefficients.

The ordinary least squares estimator minimizes the sum of squared residuals. In matrix notation, the model is expressed as:

(9) Y=Xβ

where Y is the *n* × 1 response vector, **X** is the *n* × 3 design matrix (with a leading column of ones for the intercept), and $\beta =\left(\begin{array}{c} \beta_{0}\\ \beta_{1}\\ \beta_{2} \end{array}\right)$ is the coefficient vector. The normal equations provide the closed-form solution:

(10) $$\beta ={{\left(X^{T}X\right)}^{-1}X}^{T}Y$$

This approach was applied separately to each soil dataset.

### 4.4. Performance Evaluation Criteria

To evaluate mathematical model accuracy and performance stability, four evaluation criteria are implemented:

1. For any given moisture content (MC) level, the absolute prediction error is calculated using the following equation:
  (11) $$Error\;\left(\%\right)=\left|\frac{{MC}_{predicted}-{MC}_{Actual}}{{MC}_{Actual}}\right|\times 100$$

2. MAE: Measures the average magnitude of the absolute errors between predictions and actual observations.

(12) $$MAE=\frac{1}{n}\sum_{i=1}^{n}\left|M_{i}-P_{i}\right|$$

3. RMSE: Quantifies the square root of the average squared differences, penalizing larger deviations heavily.

(13) $$RMSE=\sqrt{\frac{\sum_{i=1}^{n}{\left(P_{i}-M_{i}\right)}^{2}}{n}}$$

4. R^2^: Identifies the proportion of variance in the dependent variable that is predictable from the independent variables.

(14) $$R^{2}=1-\frac{\sum_{i=1}^{n}{\left(M_{i}-P_{i}\right)}^{2}}{\sum_{i=1}^{n}{\left(M_{i}-\bar{M}\right)}^{2}}$$

“n” is the sample size; “$M_{i}$” is the measured value for the “*ith*” observation in the dataset; “$P_{i}$” is the predicted value for the “$i^{th}$” observation in the dataset; and “$\bar{M}$” is the mean of the measured values.

### 4.5. Results

#### 4.5.1. Sandy Soil Model

**Step 1: Construct the Data Matrices (X and Y)**

To solve all seven data points simultaneously, we convert our dataset into matrices.

1. The “Y” Vector (Dependent Variable): A 7 × 1 matrix containing the actual moisture levels.
2. The “X” Matrix (Design Matrix): A 7 × 3 matrix. Crucially, we add a leading column of 1. This column of ones acts as a multiplier for the intercept ($\beta_{0}$), allowing it to be solved alongside the other coefficients.

Using the dataset, the matrices are set up like this:

$$Y=\left(\begin{array}{c} 0\\ 5\\ 10\\ 15\\ 20\\ 25\\ 30 \end{array}\right);X=\left(\begin{array}{ccc} 1 & 3.4 & 0\\ 1 & 4.5 & 0.1\\ 1 & 6.2 & 0.3\\ 1 & 8.8 & 0.6\\ 1 & 12 & 0.7\\ 1 & 15.6 & 1\\ 1 & 19.1 & 1.3 \end{array}\right)$$

And our unknown coefficients are grouped into a vector β:

$$\beta =\left(\begin{array}{c} \beta_{0}\\ \beta_{1}\\ \beta_{2} \end{array}\right)$$

**Step 2: Apply the Normal Equation Formula**

In linear algebra, we cannot directly divide matrix “Y” by matrix “X” because “X” is not a square matrix. Instead, to find the line of best fit that minimizes the sum of squared errors, we use the normal equation:

$$X^{T}X\beta =X^{T}Y$$

To isolate β, we multiply both sides by the inverse of $X^{T}X$:

$$\beta ={{\left(X^{T}X\right)}^{-1}X}^{T}Y$$

where:

$X^{T}$ is the transpose of matrix X (flipping its rows into columns).

${\left(X^{T}X\right)}^{-1}$ is the matrix inverse.

**Step 3: Compute the Matrix Transpose and Multiplications**

Calculate $X^{T}X$

We multiply the transposed matrix (3 × 7) by the original matrix (7 × 3). This yields a square 3 × 3 matrix:

$$X^{T}X=\left(\begin{array}{ccccccc} 1 & 1 & 1 & 1 & 1 & 1 & 1\\ 3.4 & 4.5 & 6.2 & 8.8 & 12 & 15.6 & 19.1\\ 0 & 0.1 & 0.3 & 0.6 & 0.7 & 1 & 1.3 \end{array}\right)\left(\begin{array}{ccc} 1 & 3.4 & 0\\ 1 & 4.5 & 0.1\\ 1 & 6.2 & 0.3\\ 1 & 8.8 & 0.6\\ 1 & 12 & 0.7\\ 1 & 15.6 & 1\\ 1 & 19.1 & 1.3 \end{array}\right)\;=\left(\begin{array}{ccc} 7 & 69.6 & 4\\ 69.6 & 899.86 & 56.42\\ 4 & 56.42 & 3.64 \end{array}\right)$$

Calculate $X^{T}Y$

Next, multiply the transposed matrix (3 × 7) by the target vector Y (7 × 1), resulting in a 3 × 1 matrix:

$$X^{T}Y=\left(\begin{array}{ccccccc} 1 & 1 & 1 & 1 & 1 & 1 & 1\\ 3.4 & 4.5 & 6.2 & 8.8 & 12 & 15.6 & 19.1\\ 0 & 0.1 & 0.3 & 0.6 & 0.7 & 1 & 1.3 \end{array}\right)\left(\begin{array}{c} 0\\ 5\\ 10\\ 15\\ 20\\ 25\\ 30 \end{array}\right)=\left(\begin{array}{c} 105\\ 1419.5\\ 90.5 \end{array}\right)$$

**Step 4: Compute the Inverse and Solve for** β

Now we must calculate the inverse of the 3 × 3 matrix, ${\left(X^{T}X\right)}^{-1}$. Using standard matrix inversion (determinants and cofactors), we get:

$${\left(X^{T}X\right)}^{-1}=\left(\begin{array}{ccc} 3.0685 & -0.9199 & 10.8871\\ -0.9199 & 0.3152 & -3.8754\\ 10.8871 & -3.8754 & 48.3799 \end{array}\right)$$

Finally, we multiply this inverse matrix by our $X^{T}Y$ vector to get the beta coefficients:

$$\beta =\left(\begin{array}{ccc} 3.0685 & -0.9199 & 10.8871\\ -0.9199 & 0.3152 & -3.8754\\ 10.8871 & -3.8754 & 48.3799 \end{array}\right)\left(\begin{array}{c} 105\\ 1419.5\\ 90.5 \end{array}\right)$$

Final Output

$$\beta =\left\{\begin{array}{c} \beta_{0}=1.6194\\ \beta_{1}=0.1752\\ \beta_{2}=20.3667 \end{array}\right.$$

The resulting regression equation is:

$$MC(\%)=1.6194+0.1752\varepsilon^{\prime }+20.3667\varepsilon^{\prime \prime }$$

The performance of the multiple linear regression model for sandy soil is summarized in Table 12, which compares the actual and predicted moisture contents at each calibration point.

Aggregate performance metrics for the sandy soil model were: MAE = 1.15%, RMSE = 1.36%, and R^2^ = 0.9814. Due to the limited number of available reference permittivity values at 1.3 GHz (seven points for sand and nine points for loam), it was not possible to reserve a sufficiently large disjoint test set. The reported MAE, RMSE, and (R^2^) values should thus be interpreted as measures of the goodness of fit of the empirical models over the calibration range, rather than as unbiased estimates of field-deployment accuracy. Future work will focus on collecting larger experimental datasets to compute independent validation statistics, including bias and confidence intervals, for different soil textures.

#### 4.5.2. Loamy Soil Model

For loam soil, the same multiple linear regression procedure described in Section 4.5.1 was applied to the loam dataset, using the simulated real and imaginary parts of permittivity as predictors and the moisture content (MC) as the response. The resulting model was then used to estimate MC for each measurement point, as summarized in Table 13. The resulting regression equation is:

$$MC(\%)={2.826-0.6119\varepsilon }^{\prime }+{19.0483\varepsilon }^{\prime \prime }$$

The performance of the multiple linear regression model for loamy soil is summarized in Table 13, which compares the actual and predicted moisture contents at each calibration point.

Aggregate performance metrics for the loamy soil model were: MAE = 1.22%, RMSE = 1.58%, and R^2^ = 0.985.

## 5. Experimental Prototype and Validation

### 5.1. Sensor Fabrication

The proposed square SRR-based sensor was fabricated using standard PCB technology on Rogers RO3010 substrate with thickness = 1.27 mm, and 35 µm copper cladding on both sides. The layout strictly follows the optimized dimensions in Table 1 to preserve the designed resonance characteristics and field confinement in the sensing region. Surface mount connectors were used to interface the input and output ports with the vector network analyzer (VNA_ E5071B).

**Figure 13:**
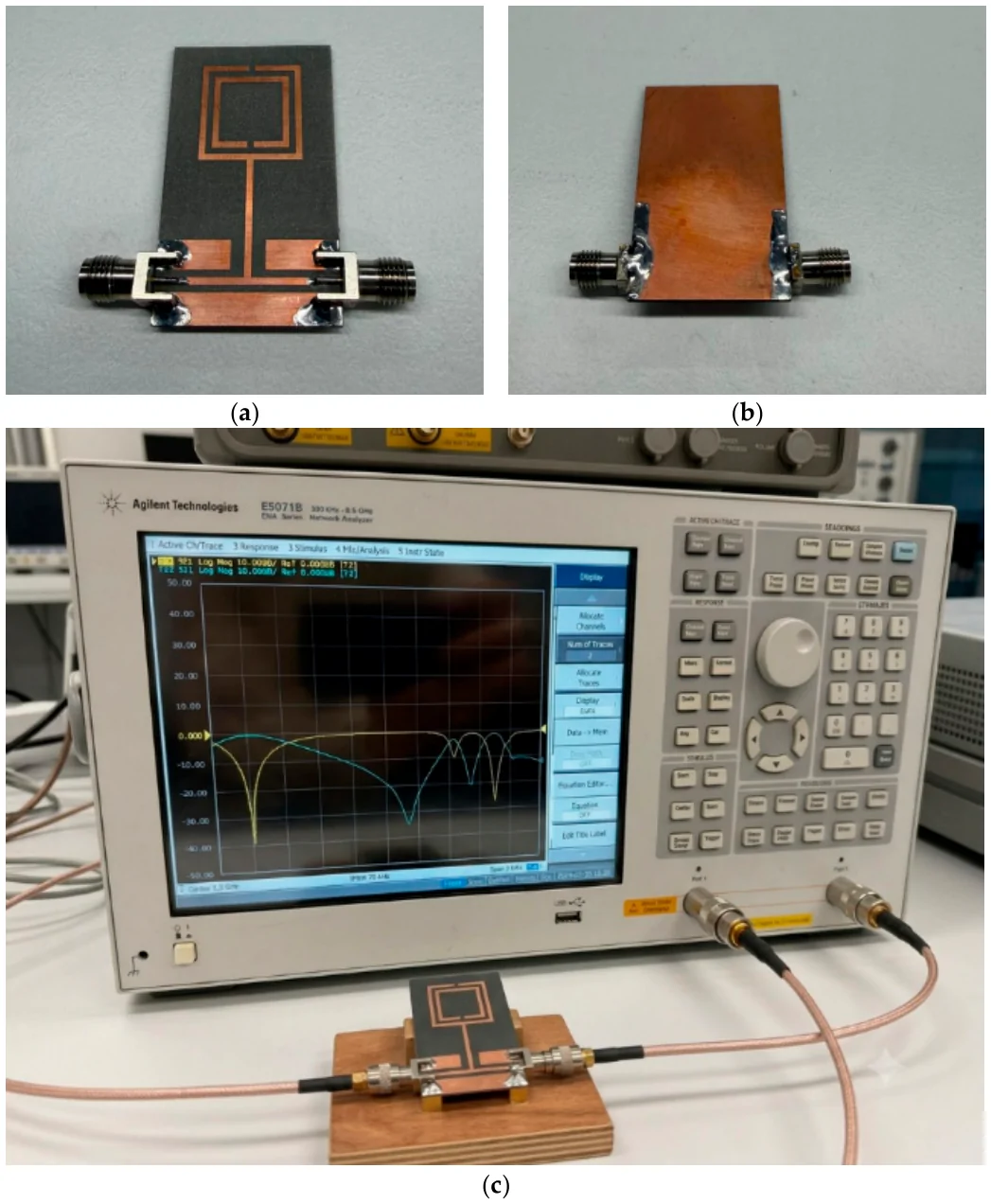
Photographs of the fabricated square SRR-based soil moisture sensor prototype: (**a**) top view showing the square SRR and T-shaped feedline, (**b**) bottom view of the ground plane, and (**c**) measurement setup with VNA.

**Figure 14:**
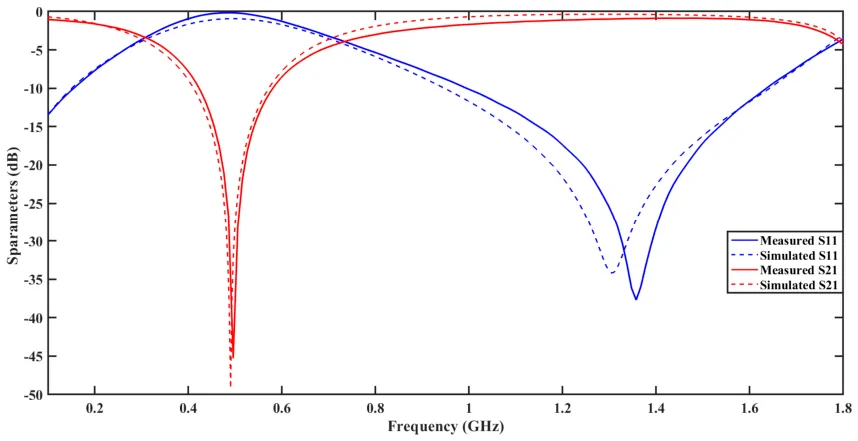
Simulated and measured |S_11_| and |S_21_| response of the proposed square SRR-based sensor.

Figure 13 shows photographs of the fabricated prototype, including (a) the top view with the square SRR and T-shaped feedline, (b) the bottom ground plane, and (c) a close-up of the sensing region. The measured and simulated |S11| and |S21| responses of the unloaded sensor (without soil) are compared in Figure 14. The fabricated device exhibits a clear resonance around 1.3 GHz, in good agreement with simulations, with minor deviations in resonance frequency and insertion loss that can be attributed to fabrication tolerances, connector parasitic, and slight discrepancies in the actual substrate permittivity and loss tangent.

### 5.2. Measurement Setup

Two soil types were selected for experimental validation: sandy soil and loamy soil. Bulk soil samples were first air-dried at room temperature, gently crushed, and sieved to remove stones and large organic debris, ensuring homogeneous packing in the soil box. The gravimetric moisture content (MC) was adjusted by adding predetermined amounts of water to the dry soil. For each target MC level, the soil mass before and after water addition was recorded using a precision balance (±0.0001 g) to verify the actual moisture content. The conditioned samples were then sealed in airtight containers and allowed to rest for several hours to promote uniform redistribution of water prior to measurement.

To reduce the influence of bulk-density variations on the effective dielectric properties, all soil samples were packed using a consistent procedure. For each moisture treatment, the soil was gently poured into the container in thin layers and the container was lightly tapped on the bench to achieve a reproducible compaction level without over-consolidation. The total mass of soil placed in the box at each moisture level was kept approximately constant across repetitions, and care was taken not to disturb the packing when positioning the container on the sensor. This procedure limits changes in porosity and bulk density, so that the observed resonance frequency shifts can be primarily attributed to moisture-induced permittivity changes rather than packing artifacts.

**Figure 15:**
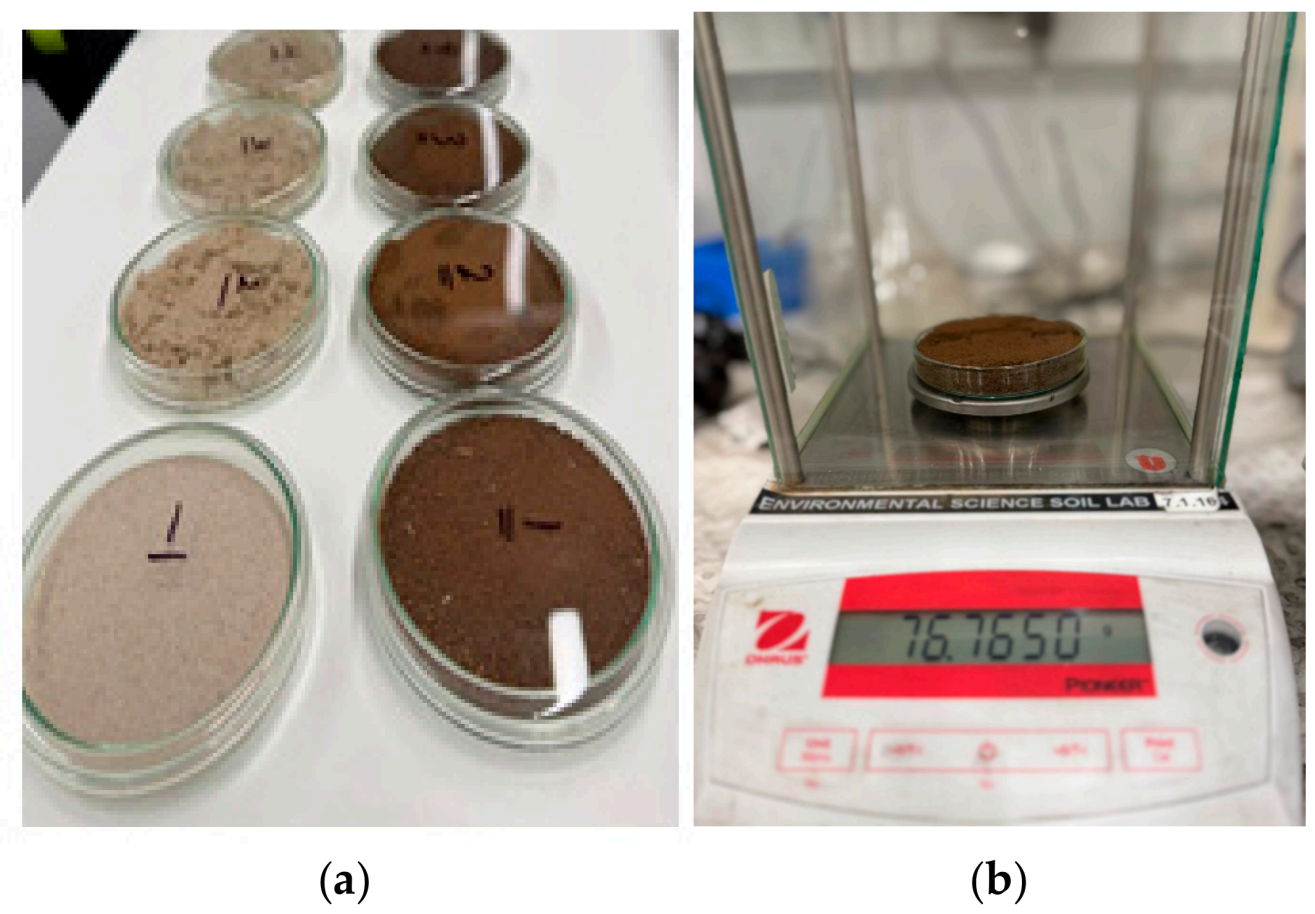
Soil preparation: (**a**) soil samples, and (**b**) gravimetric moisture content procedure.

The soil samples were prepared and their gravimetric moisture content was determined following the procedure illustrated in Figure 15.

**Figure 16:**
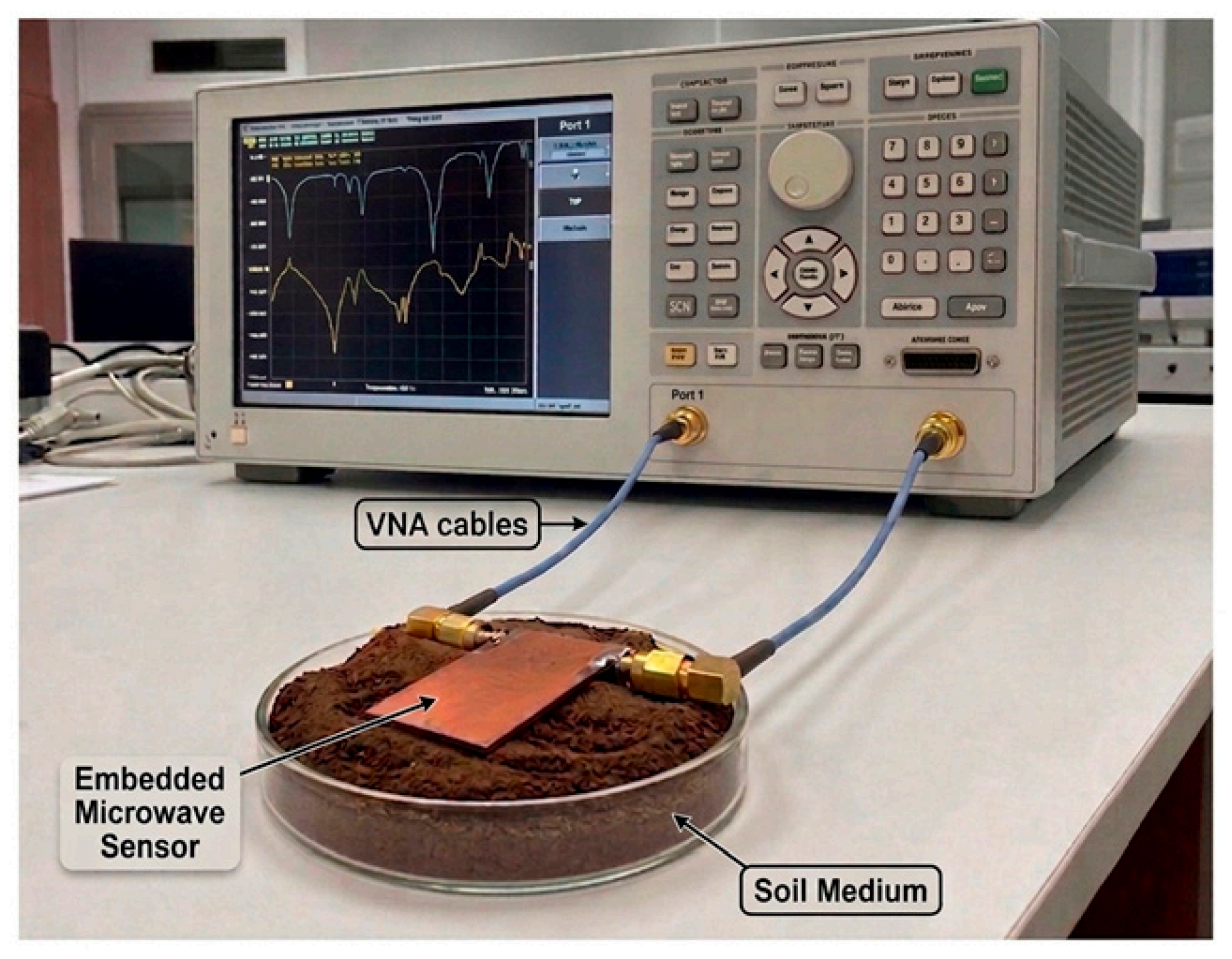
Measurement setup of the sensor with the soil.

To ensure reproducible interaction between the electromagnetic field and the Soil Under Test (SUT), a dedicated soil container was used as an under the SRR, as shown in Figure 16.

**Figure 17:**
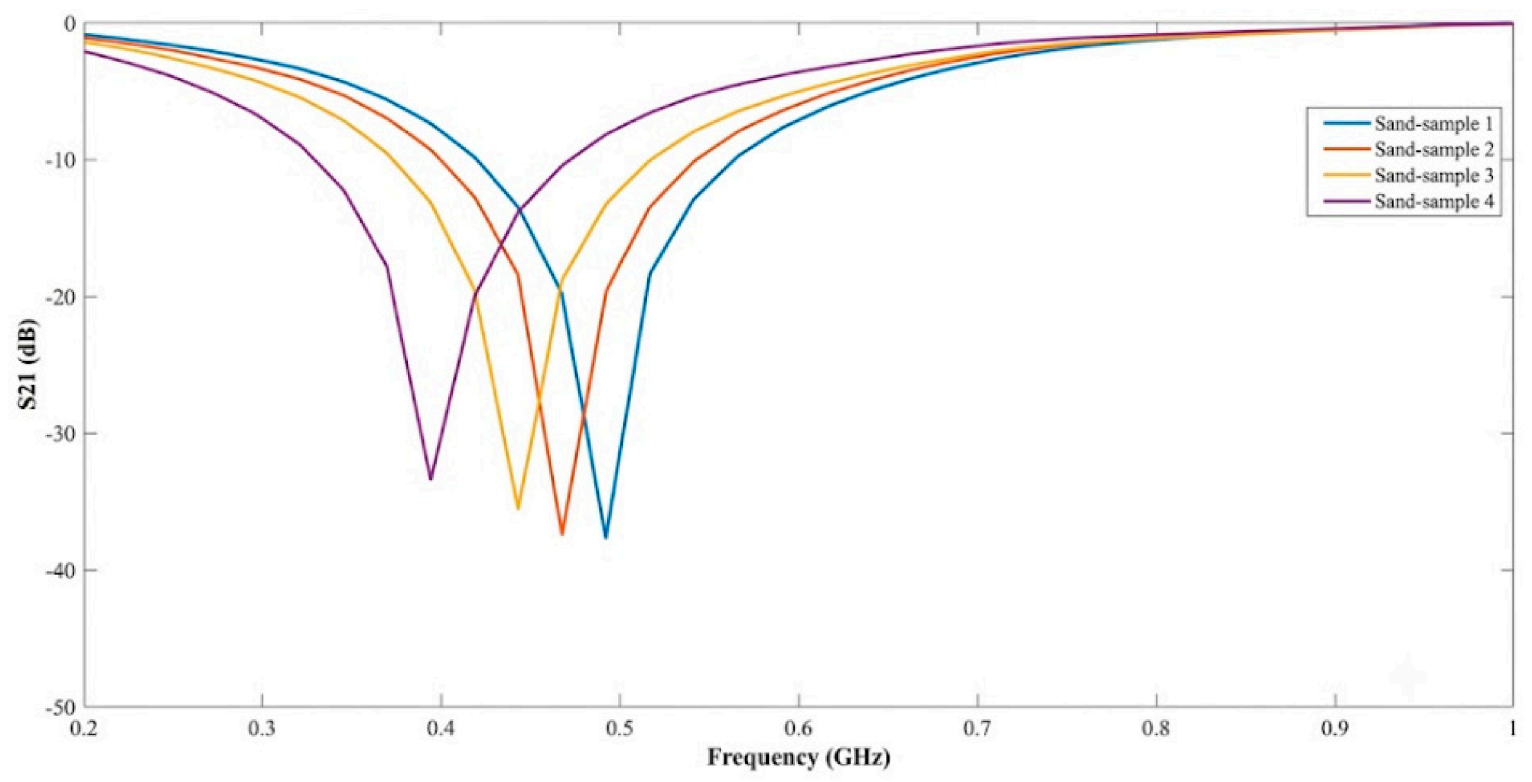
Measured S_21_ response of square SRR-based sensor with sandy soil.

The measurement procedure comprised the following steps. First, subsamples of dry soil were precisely weighed and the required amount of water was added to reach the desired MC level. The moist soil was placed into labeled containers and its frequency response (resonance frequency and insertion loss) was measured using the proposed sensor integrated into a stabilized holder to minimize movement during VNA measurements. After the microwave measurements, each sample was transferred to a preheated oven (Figure 17) and dried at 105–110 °C for 24 h to determine the final dry mass.

The gravimetric moisture content was then calculated using:

(15) $$MC\left(\%\right)=\frac{m_{water}}{m_{DrySoil}}\times 100\%=\frac{M_{2}-M_{3}}{M_{3}-M_{1}}\times 100\%$$

where M_1_ is the mass of the empty container, M_2_ is the mass of the container plus moist soil, and M_3_ is the mass of the container plus oven-dried soil.

Potential sources of measurement uncertainty include VNA calibration drift, connector repeatability, slight misalignments between the soil box and the sensor, variability in soil packing and bulk density, and tolerances in the substrate permittivity and loss tangent. To mitigate these effects, a full two-port SOLT calibration was performed before each measurement session, and the sensor together with the soil container was mounted in a rigid holder to maintain consistent positioning. For each moisture content, the S-parameters were measured multiple times after re-packing the soil, and the resonance frequency and (S21) at resonance were extracted from the average response.

This protocol was applied to establish experimental calibration points for both sandy and loamy soils. The measured weights and corresponding moisture contents are summarized in Table 14 and Table 15.

### 5.3. Measured Soil-Loaded Response and Model Validation

**Figure 18:**
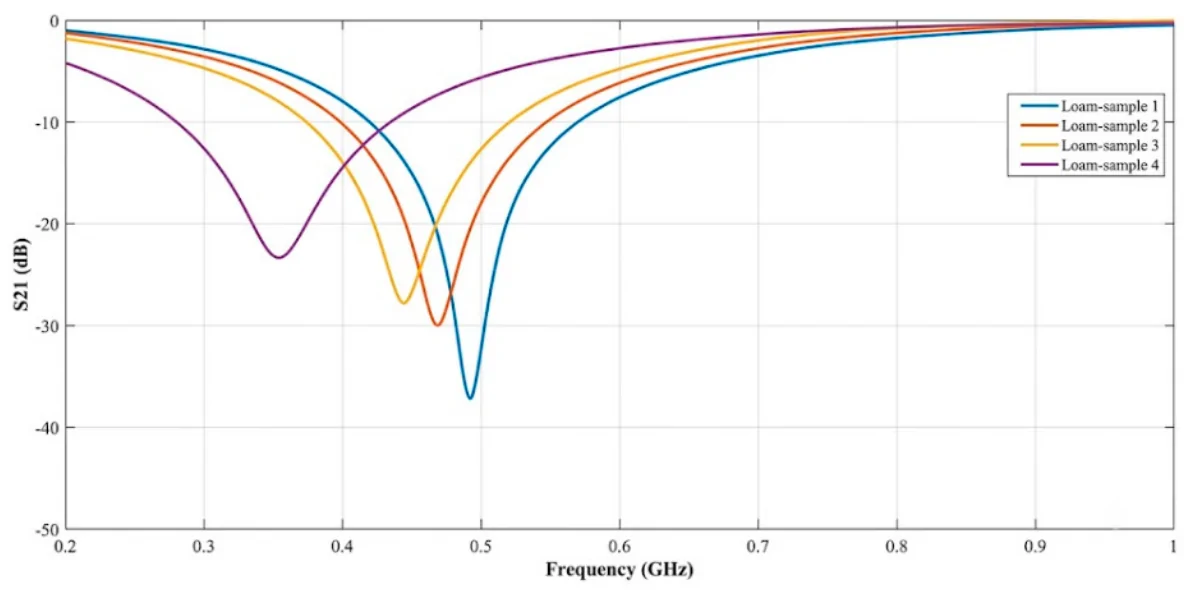
Measured S_21_ response of square SRR-based sensor with loamy soil.

The measured transmission responses (S21) of the square SRR-based sensor loaded with sandy and loamy soils at different moisture contents are shown in Figure 17 and Figure 18, respectively. For each soil type, four representative moisture groups were considered, spanning practical agricultural ranges. The corresponding resonance frequencies, insertion losses, and gravimetrically determined moisture contents are listed in Table 16 for sandy soil and Table 17 for loamy soil.

For sandy soil (Figure 17 and Table 16), the measured moisture groups cover approximately 4.3–30.1% MC, whereas for loamy soil (Figure 18 and Table 17), the measurements span approximately 1.1–20.5% MC. In both cases, increasing MC produces a systematic shift in the resonance frequency toward lower values accompanied by moderate changes in (|S21|) at resonance. These experimental ranges are narrower than the simulated calibration ranges of 0–30% for sand and 0–40% for loam considered in Section 4. Consequently, the experimental results provide partial validation of the sensor response and calibration trends over practical agricultural moisture levels but do not yet constitute full experimental coverage of the entire calibration domain, particularly at very low and very high moisture contents. Extending the measurements to additional moisture levels and soil batches is planned as future work to close this gap.

The comparison between Table 3 and Table 4 (simulated performance) and Table 16 and Table 17 (measured performance) demonstrates that the fabricated sensor preserves the moisture sensitivity predicted by the numerical analysis. The measured frequency shifts across the tested MC ranges are comparable to the simulated ~115 MHz span reported earlier, confirming the robustness of the sensor’s resonance behavior under practical loading conditions. These experimental data can be directly used with the polynomial and machine-learning-based calibration models developed in Section 4 by feeding the measured resonance frequency and S21 magnitude as inputs, thereby enabling estimation of soil permittivity and MC from real sensor measurements. The close qualitative agreement between simulated (Figure 8 and Figure 9, Table 3 and Table 4) and measured responses (Figure 17 and Figure 18, Table 16 and Table 17) validates the proposed square SRR-based sensor as a reliable platform for soil moisture characterization.

To assess measurement repeatability, each soil–moisture condition in Table 16 and Table 17 was measured multiple times after re-packing the soil. For each group, the resonance frequency and (|S21|) at resonance were extracted and summarized as mean ± standard deviation. Across all moisture groups, the standard deviation of the resonance frequency remained small compared to the total observed frequency span, and the variation in (|S21|) at resonance was within a narrow interval. These results indicate that, under the present laboratory conditions and packing procedure, the proposed sensor exhibits stable and repeatable responses suitable for calibration and moisture estimation.

## 6. Discussion

### 6.1. Regression Model

The proposed square SRR-based sensor demonstrates a reliable and interpretable relationship between soil moisture content and the sensor’s resonance frequency shift. Both polynomial curve fitting and multiple linear regression models confirm that the sensor’s frequency displacement effectively reflects changes in the soil’s real permittivity, which correlates with moisture variation. The machine learning calibration model enhances this interpretation by integrating frequency and transmission loss parameters, improving prediction accuracy across sandy and loamy soils. The regression coefficients explicitly link the real and imaginary components of soil permittivity to moisture content, enabling quantitative differentiation of soil types and moisture levels. The slightly better model performance for sandy soil aligns with its simpler dielectric behavior due to lower bound water content and surface area compared to loam.

### 6.2. Error Analysis

The sensor models exhibit strong predictive accuracy, with low mean absolute error (MAE) and root mean square error (RMSE), alongside high coefficients of determination (R^2^ > 0.98) for both soil types. For sandy soil, MAE and RMSE are 1.15% and 1.36%, respectively, while for loamy soil they are 1.22% and 1.58%, respectively. These error margins are within acceptable limits for practical soil moisture monitoring. The observed resonance frequency shifts, reaching up to approximately 115 MHz over the moisture range, demonstrate the sensor’s sensitivity and robustness. The consistent downward frequency shift with increasing moisture content validates the sensor’s responsiveness to dielectric property changes, confirming its suitability for precise moisture quantification.

### 6.3. Comparison with the Literature

The overall calibration process adopted in this work consists of two stages. First, a machine-learning-based calibration model is trained to estimate the real and imaginary parts of the soil permittivity (ε′, ε″) from the sensor’s electromagnetic response (resonance frequency shift and transmission loss S21). This model is fitted using the simulated datasets in Table 2, Table 3 and Table 4, for which ε′ and ε″ are known, and subsequently used to infer ε′ and ε″ from the measured S-parameters of the sensor. In the second stage, the soil moisture content (MC) is obtained from the estimated permittivity values using a multiple linear regression (MLR) model of Equation 8.

Unlike several recent planar microwave soil moisture sensors that operate at higher frequencies (typically above 2 GHz) with reduced penetration depth and often rely on single-feature calibration (usually resonance frequency only) or validation on a single soil texture, the proposed sensor combines low-frequency operation (1.3 GHz), compact geometry (26 × 43 mm^2^), and multi-feature, texture-specific calibration. As summarized in Table 18, this enables us to address specific limitations in [30,31,32,33] in terms of operating depth, footprint, and calibration robustness.

In particular, the proposed square SRR sensor offers deeper field penetration than higher-frequency designs in [30,31,32], while occupying a smaller or comparable footprint. The maximum sensitivity of 3.4% is higher than that reported in [30] and competitive with other resonator-based sensors where sensitivity is not explicitly quantified. Moreover, by developing separate calibration models for sandy and loamy soils, and by incorporating both resonance frequency shifts and transmission loss into polynomial, machine-learning, and multiple-regression models, the present work explicitly accounts for soil texture and complex permittivity components, whereas most existing studies focus on a single soil type and a single calibration feature. These differences translate into practical benefits for texture-aware soil moisture estimation in agricultural and environmental applications.

The proposed square SRR-based sensor therefore shows competitive advantages over other microwave soil moisture sensors, as detailed in Table 18. Operating at a lower frequency of 1.3 GHz, it offers enhanced soil penetration depth compared with designs around 2.38–2.46 GHz, while its compact size of 26 × 43 mm is smaller than or comparable to sensors such as the 50 × 50 mm design in [30], the 30 × 40 mm sensor in [31], and the 96 × 46 mm antenna sensor in [32]. The measured maximum sensitivity of 3.4% exceeds the 1.83% reported in [30] and remains competitive where sensitivity is not specified. In addition, validation on both sandy and loamy soils over agriculturally relevant ranges (0–30% and 0–40% simulated; ≈4–30% and ≈1–20% experimentally) and the use of explicit, closed-form data-driven calibration models distinguish the present work from many earlier studies that address only a single soil type or rely on simpler calibration curves. Overall, the sensor delivers a well-balanced combination of low operating frequency, compact footprint, high sensitivity, and texture-aware calibration, positioning it as a strong candidate among current microwave soil moisture sensing solutions.

### 6.4. Limitations and Future Work

The present study has several limitations that should be acknowledged. First, the calibration models are primarily based on literature-derived dielectric properties and simulated S-parameter responses at 1.3 GHz, while the experimental validation is restricted to a relatively small number of moisture groups and soil batches. Second, only two soil textures (sandy and loamy) were considered, and the proposed models may require re-calibration or adaptation for clay-rich or highly organic soils. Third, all measurements were conducted under controlled laboratory conditions, and field-scale factors such as temperature variations, salinity, and heterogeneous layering were not explicitly studied. Addressing these limitations will require (i) extending the experimental dataset to more moisture levels, soil types, and sampling locations, (ii) refining the calibration models with larger training and independent test sets, and (iii) performing field deployments to evaluate long-term stability and robustness under realistic agricultural and environmental conditions.

## 7. Conclusions

The proposed square split ring resonator (SRR)-based sensor demonstrates effective design and simulation outcomes for soil moisture content characterization at 1.3 GHz. The compact microstrip sensor, developed on Rogers RO3010 substrate, exhibits strong sensitivity to moisture-induced changes in soil permittivity, with a measurable resonance frequency shift of approximately 115 MHz across practical moisture ranges for sandy (0–30%) and loamy (0–40%) soils. Calibration models employing polynomial curve fitting, machine learning, and multiple linear regression confirm high predictive accuracy, with MAE below 1.22%, RMSE under 1.58%, and R^2^ exceeding 0.98 for both soil types. The sensor’s lower operating frequency enhances soil penetration depth compared to existing microwave sensors, while its compact size and high sensitivity facilitate integration into agricultural monitoring systems. These results validate the sensor’s potential as a reliable, cost-effective tool for precise, non-contact soil moisture measurement, offering advantages in sensitivity and practical applicability over higher-frequency planar alternatives. Future work will focus on extended experimental validation under field conditions, evaluation across additional soil textures, and refinement of the calibration framework for real-time in situ deployment.

## Figures and Tables

**Table 1:** Parameters of designed square SRR-based sensor.

Symbol	Description	Value (mm)
**h_Sub_**	Height of substrate	1.27
**t**	Thickness of SRR	0.035
**W_sub_**	Substrate total width (height)	26
**L_sub_**	Substrate total length	43
**W**	CPW feedline signal width	1.00
**S__cpw_**	CPW gap spacing	1.00
**Wgnd_cpw**	CPW ground plane width	4.20
**W__conn_**	Width of quarter-wavelength	1.00
**λ/4**	Quarter-wavelength	17.13
**L_1_**	Inner ring side length	12.00
**L_2_**	Outer ring side length	16.00
**W__ring_**	Split-ring trace width	1.00
**S__ring_**	Spacing between inner and outer rings	1.00
**g**	Split-ring capacitive gap	1.00

**Table 2:** Dielectric properties of sandy and loamy soil [27].

Soil Type	Soil Moisture (%)	Real Part (ε′)	Imaginary Part (ε″)
**Sand**	0	3.4	0
	5	4.5	0.1
	10	6.2	0.3
	15	8.8	0.6
	20	12.0	0.7
	25	15.6	1.0
	30	19.1	1.3
**Loam**	0	3.4	0.0
	5	4.5	0.3
	10	6.0	0.6
	15	7.6	0.9
	20	9.8	1.2
	25	12.6	1.5
	30	16.5	1.8
	35	20.5	2.3
	40	24.5	2.9

**Table 3:** Performances of designed square SRR-based sensor with sandy soil.

Moisture Level (MC)	$\varepsilon^{\prime }$ Dataset [27]	$\varepsilon^{\prime \prime }$ Dataset [27]	$\Delta f_{notch}$ (GHz) Simulated	$f_{notch}$ (GHz) Simulated	S_21_ (dB) Simulated	$S_{n}$ (%)
0%	3.4	0.0	0.03888	0.43732	49.799	3.4
5%	4.5	0.1	0.05184	0.42436	41.488	3.11
10%	6.2	0.3	0.06989	0.40631	35.493	2.82
15%	8.8	0.6	0.09056	0.38564	32.181	2.43
20%	12	0.7	0.11366	0.36254	31.115	2.16
25%	15.6	1.0	0.13396	0.34224	30.113	1.92
30%	19.1	1.3	0.14938	0.32682	29.616	1.73

**Table 4:** Performances of designed square SRR-based sensor with loamy soil.

Moisture Level (MC)	$\varepsilon^{\prime }$ Dataset [27]	$\varepsilon^{\prime \prime }$ Dataset [27]	$\Delta f_{notch}$ (GHz) Simulated	$f_{notch}$ (GHz) Simulated	S_21_ (dB) Simulated	$S_{n}$(%)
0%	3.4	0.0	0.03888	0.43732	49.799	3.4019
5%	4.5	0.3	0.05206	0.42414	35.682	3.1235
10%	6.0	0.6	0.0687	0.4075	31.899	2.8853
15%	7.6	0.9	0.08241	0.39379	29.179	2.6221
20%	9.8	1.2	0.09799	0.37821	27.258	2.3384
25%	12.6	1.5	0.11727	0.35893	26.901	2.1229
30%	16.5	1.8	0.14106	0.33514	26.122	1.9111
35%	20.5	2.3	0.1596	0.3166	25.496	1.7187
40%	24.5	2.9	0.1759	0.3003	24.518	1.5718

**Table 5:** Polynomial fitting model results.

Moisture Level (MC)	$\varepsilon^{\prime }$ Dataset	$\Delta f_{notch}$ (GHz) Simulated	$\varepsilon^{\prime }$ Simulated	Error (%)
0%	3.4	0.03888	3.4907	2.6665
5%	4.5	0.05184	4.4450	1.2211
10%	6.2	0.06989	6.1538	0.74454
15%	8.8	0.09056	8.6534	1.6659
20%	12	0.11366	12.132	1.1038
25%	15.6	0.13396	15.787	1.2004
30%	19.1	0.14938	18.937	0.85346

**Table 6:** Polynomial fitting model results.

Moisture Level (MC)	$\varepsilon^{\prime }$ Dataset	$\Delta f_{notch}$ (GHz) Simulated	$\varepsilon^{\prime }$ Simulated	Error(%)
0%	3.4	0.03888	3.4786	2.313
5%	4.5	0.05206	4.4683	0.706
10%	6.0	0.0687	6.0259	0.432
15%	7.6	0.08241	7.5678	0.424
20%	9.8	0.09799	9.6034	2.006
25%	12.6	0.11727	12.540	0.476
30%	16.5	0.14106	16.800	1.819
35%	20.5	0.1596	20.608	0.525
40%	24.5	0.1759	24.308	0.784

**Table 7:** Soil type standardization parameters for sand and loam soils.

Soil Types	Standardization
**Sand**	- Values for ${\Delta f}_{soil}$: Mean μ=0.00124 and scale σ=0.03504 - Values for $\Delta S_{21}$: Mean μ=3.52429 and scale σ=7.10599 ${x1}_{norm}=\frac{\Delta f-0.00124}{0.03504};$ ${x2}_{norm}=\frac{\Delta S_{21}-3.52429\;}{7.10599}$
**Loam**	- Values for ${\Delta f}_{soil}$: Mean μ=−0.00578 and scale σ=0.04533 - Values for $\Delta S_{21}$: Mean μ=3.50356 and scale σ=7.497 ${x1}_{norm}=\frac{\Delta f+0.00578}{0.04533};$ ${x2}_{norm}=\frac{\Delta S_{21}-3.50356\;}{7.497}$

**Table 8:** Soil type network equations (sandy and loamy soils).

Soil Types	Network Equations
Sand	$h_{1}=tanh((-4.0958\;*\;{x1}_{norm})+(4.9316\;*\;{x2}_{norm})+(-0.8965))$ $h_{2}=tanh((-1.4562\;*\;{x1}_{norm})+(0.3813\;*\;{x2}_{norm})+(1.1019))$ $h_{3}=tanh((-1.0977\;*\;{x1}_{norm})+(-1.9426\;*\;{x2}_{norm})+(2.8993))$ $h_{4}=tanh((10.8675\;*\;{x1}_{norm})+(1.1899\;*\;{x2}_{norm})+(5.9188))$ $h_{5}=tanh((0.4764\;*\;{x1}_{norm})+(-3.1216\;*\;{x2}_{norm})+(4.1275))$
Loam	$h_{1}=tanh((0.1135\;*\;{x1}_{norm})+(-0.6629\;*\;{x2}_{norm})+(3.3497))$ $h_{2}=tanh((1.2208\;*\;{x1}_{norm})+(1.6740\;*\;{x2}_{norm})+(1.4703))$ $h_{3}=tanh((0.8433\;*\;{x1}_{norm})+(0.0147\;*\;{x2}_{norm})+(-0.2652))$ $h_{4}=tanh((1.6946\;*\;{x1}_{norm})+(0.0788\;*\;{x2}_{norm})+(2.4115))$ $h_{5}=tanh((-1.7643\;*\;{x1}_{norm})+(0.9760\;*\;{x2}_{norm})+(-4.2915))$

**Table 9:** Soil type prediction equations (sandy and loamy soils).

Soil Types	Prediction Equations
Sand	$\varepsilon^{\prime }=\left(8.1034*h_{1}\right)+\left(4.7685*h_{2}\right)+\left(2.4141*h_{3}\right)-(0.8939*h_{4})+(6.7725*h_{5})+5.0258$ $\varepsilon^{\prime \prime }=(0.6862*h_{1})+(0.5542*h_{2})+(0.2043*h_{3})+(0.0292*h_{4})+(0.5768*h_{6})+0.06$
Loam	$\varepsilon^{\prime }=\left(4.3042*h_{1}\;\right)-\left(1.3702*h_{2}\;\right)-\left(6.2179*h_{3}\;\right)-\left(5.9034*h_{4}\;\right)-(5.4255*h_{5})+5.8418$ $\varepsilon^{\prime \prime }=\left(2.5223*h_{1}\right)+\left(0.3052*h_{2}\right)-\left(1.2690*h_{3}\right)-\left(0.9244*h_{4}\right)+\left(0.4164*h_{5}\right)-0.4048$

**Table 10:** Machine learning model results for sandy soil.

Moisture Level (MC)	Δf (GHz)	${\Delta S}_{21}$ (dB)	$\varepsilon^{\prime }$ Predicted	$\varepsilon^{\prime \prime }$ Predicted	Error (%) $\varepsilon^{\prime }$	Error (%) $\varepsilon^{\prime \prime }$
0%	0.05168	17.618	3.4009	0.0001	0.03	N/A
5%	0.03872	9.307	4.4998	0.1000	0.00	0.02
10%	0.02067	3.312	6.1998	0.3000	0.00	0.01
15%	0.00000	0.000	8.7986	0.5998	0.02	0.03
20%	−0.02310	−1.066	11.9962	0.6991	0.03	0.13
25%	−0.04340	−2.068	15.5994	1.0016	0.00	0.16
30%	−0.05882	−2.565	19.0998	1.2992	0.00	0.06

**Table 11:** Machine learning model results for loamy soil.

Moisture Level (MC)	Δf (GHz)	${\Delta S}_{21}$ (dB)	$\varepsilon^{\prime }$ Predicted	$\varepsilon^{\prime \prime }$ Predicted	Error (%) $\varepsilon^{\prime }$	Error (%) $\varepsilon^{\prime \prime }$
0%	0.05911	22.541	3.3999	0.0001	0.00	N/A
5%	0.04593	8.424	4.5002	0.2989	0.00	0.38
10%	0.02929	4.641	5.9998	0.6028	0.00	0.47
15%	0.01558	1.921	7.6000	0.8975	0.00	0.28
20%	0	0	9.8001	1.2007	0.00	0.06
25%	−0.01928	−0.357	12.5998	1.5003	0.00	0.02
30%	−0.04307	−1.136	16.5000	1.7994	0.00	0.03
35%	−0.06161	−1.762	20.4998	2.3005	0.00	0.02
40%	−0.07791	−2.74	24.4996	2.8998	0.01	0.01

**Table 12:** Sandy soil: Actual versus predicted moisture content.

MC Actual	$\varepsilon^{\prime }$	$\varepsilon^{\prime \prime }$	MC Predicted	Error (%)
0%	3.4	0.0	2.21%	N/A
5%	4.5	0.1	4.44%	11.11
10%	6.2	0.3	8.81%	11.84
15%	8.8	0.6	15.38%	2.54
20%	12	0.7	17.97%	10.10
25%	15.6	1.0	24.71%	1.12
30%	19.1	1.3	31.44%	4.8

**Table 13:** Loamy soil: Actual versus predicted moisture content.

MC Actual	$\varepsilon^{\prime }$	$\varepsilon^{\prime \prime }$	MC Predicted	Error (%)
0%	3.4	0.0	0.745	N/A
5%	4.5	0.3	5.787	15.742
10%	6.0	0.6	10.583	5.838
15%	7.6	0.9	15.319	2.129
20%	9.8	1.2	19.687	1.561
25%	12.6	1.5	23.689	5.243
30%	16.5	1.8	27.017	9.942
35%	20.5	2.3	34.094	2.588
40%	24.5	2.9	43.075	7.689

**Table 14:** Gravimetric moisture content determination for sandy soil.

Moisture Content Group	Weight of Can, M_1_ (g)	Weight of Can + Wet Soil, M_2_ (g)	Weight of Can + Dry Soil, M_3_ (g)	Calculated MC (%)
1	29.2465	78.9756	77.1721	3.7631
2	29.2979	84.0430	77.2287	14.2169
3	30.8744	89.4140	78.3338	23.3467
4	29.2086	91.6390	77.1023	30.3520

**Table 15:** Gravimetric moisture content determination for loamy soil.

Moisture Content Group	Weight of Can, M_1_ (g)	Weight of Can + Wet Soil, M_2_ (g)	Weight of Can + Dry Soil, M_3_ (g)	Measured MC (%)
1	29.6131	79.5762	79.5530	0.0465
2	29.1885	84.1115	79.3093	9.5813
3	29.6892	86.9140	79.5970	14.6610
4	29.1819	87.3750	77.8857	19.4837

**Table 16:** Measured performances of the fabricated square SRR-based sensor with sandy soil.

Moisture Content Group	$\Delta f_{notch}$ (GHz) Measured	$f_{notch}$ (GHz) Measured	Δ S_21_ (dB) Measured	S_21_ (dB) Measured	Measured MC (%)
1	0.004	0.492	7.58	37.7	4.3
2	0.029	0.467	7.78	37.5	13.9
3	0.053	0.443	9.68	35.6	22.6
4	0.102	0.394	11.88	33.4	30.1

**Table 17:** Measured performances of the fabricated square SRR-based sensor with loamy soil.

Moisture Content Group	$\Delta f_{notch}$ (GHz) Measured	$f_{notch}$ (GHz) Measured	ΔS_21_ (dB) Measured	S_21_ (dB) Measured	Measured MC (%)
1	0.004	0.492	8.08	37.2	1.1
2	0.029	0.467	14.98	30.3	10.2
3	0.053	0.443	17.38	27.9	14.8
4	0.151	0.345	22.48	22.8	20.5

**Table 18:** Comparison of sensors’ performances.

Ref.	Configuration	Substrate	Operating Frequency (GHz)	Size (mm^2^)	Sensitivity	Soil Type	Moisture Content Range
[31]	DGS-based planar sensor (mod. CSRR)	Rogers RO3006	2.38	50 × 50	1.83%	Fine sand Coarse sand Grit Clay	7 to 15%
[32]	Self-similar fractal planar sensor (MPS)	FR4	2.4	30 × 30	Not specified	Soil with organic matter content	0–100%
[33]	Cavity antenna (planar)	Not specified	1.5–3	96 × 46	Not specified	Silty, clay, and loam	1–45%
	Rotated self-similar fractal (R-SSF) planar sensor	Rogers RT 5880	2.4	60 × 50	Not specified	Sandy, loam, clayey	0–75% field capacity
This work	Square SRR sensor	Rogers RO3010	1.3	26 × 43	3.4%	Sand Loam	0–30% 0–40%

## Data Availability

The data used and analyzed during the current study available from the corresponding author on reasonable request.
